# Membrane-wrapped nanoparticles for photothermal cancer therapy

**DOI:** 10.1186/s40580-022-00328-4

**Published:** 2022-08-12

**Authors:** Sara B. Aboeleneen, Mackenzie A. Scully, Jenna C. Harris, Eric H. Sterin, Emily S. Day

**Affiliations:** 1 Biomedical Engineering, University of Delaware, Newark, DE USA; 2 Materials Science and Engineering, University of Delaware, Newark, DE USA; 3 Center for Translational Cancer Research, Helen F. Graham Cancer Center and Research Institute, Newark, DE USA

**Keywords:** Phototherapy, Multimodal therapy, Targeting, Biomimicry, Biomimetic, Nanomedicine, Oncology

## Abstract

Cancer is a global health problem that needs effective treatment strategies. Conventional treatments for solid-tumor cancers are unsatisfactory because they cause unintended harm to healthy tissues and are susceptible to cancer cell resistance. Nanoparticle-mediated photothermal therapy is a minimally invasive treatment for solid-tumor cancers that has immense promise as a standalone therapy or adjuvant to other treatments like chemotherapy, immunotherapy, or radiotherapy. To maximize the success of photothermal therapy, light-responsive nanoparticles can be camouflaged with cell membranes to endow them with unique biointerfacing capabilities that reduce opsonization, prolong systemic circulation, and improve tumor delivery through enhanced passive accumulation or homotypic targeting. This ensures a sufficient dose of photoresponsive nanoparticles arrives at tumor sites to enable their complete thermal ablation. This review summarizes the state-of-the-art in cell membrane camouflaged nanoparticles for photothermal cancer therapy and provides insights to the path forward for clinical translation.

## Introduction

### Overview of photothermal therapy (PTT) as a cancer treatment strategy

Cancer is a global health problem that needs effective treatment strategies. It is the second leading cause of death in the United States where over 600,000 people are expected to die from cancer in 2022 [1]. Conventional treatments for solid-tumor cancers include surgical resection, radiotherapy, and chemotherapy, which are often used in combination. While surgery may work against early-stage disease, in most cases it fails to remove all cancer cells, leading to recurrence. Radiotherapy and chemotherapy also face limitations as both can unintentionally harm healthy tissues and encounter cancer cell resistance [2]. There is an urgent clinical need for potent and precise treatments that can destroy solid-tumor cancers while minimizing off-target effects.

**Scheme 1:**
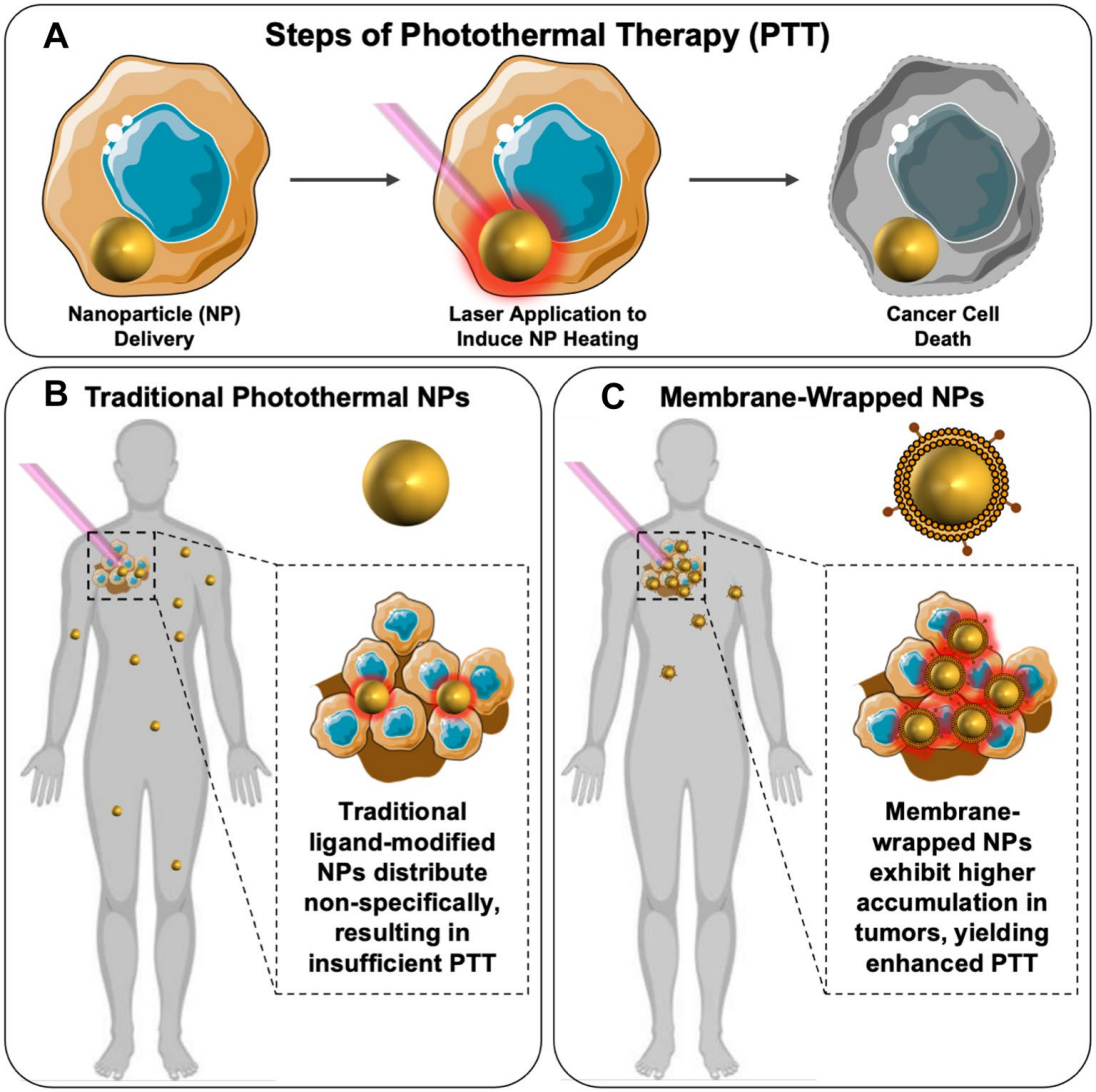
**A** Photothermal therapy involves the delivery of NPs into tumors, followed by laser irradiation to induce NP heating leading to cancer cell death. **B** Traditional PEG- or ligand-modified NPs distribute non-specifically throughout the body, and low tumor accumulation may lead to insufficient PTT effects. **C** Coating photoresponsive NPs with cell-derived membranes enhances their tumor accumulation, increasing the efficacy of PTT upon light irradiation. Portions of this figure were produced using Servier Medical Art templates (https://smart.servier.com). Servier Medical Art by Servier is licensed under a Creative Commons Attribution 4.0 Unported License

Nanoparticle-mediated photothermal therapy (PTT) is a non-invasive treatment method with immense promise as a standalone or adjuvant therapy for solid-tumor cancers. In PTT, light-responsive nanoparticles (NPs) are intravenously (IV) administered and accumulate in the tumor site, which is then irradiated with an externally applied laser tuned to match the peak plasmon resonance/absorbance wavelength of the NPs. The light irradiation causes the NPs to produce heat that irreversibly damages cancer cells in the surrounding tumor tissue (Scheme 1A, B) [3]. Apart from killing the primary tumor, under the right conditions PTT can induce an anti-tumor immunological effect that can prevent or treat metastasis and/or reduce recurrence through the release of tumor-associated antigens by the ablated tumor cells [4]. A major benefit of PTT is its high precision, as thermal ablation is achieved only at the tumor site where NPs and light are combined. Hence, off-target effects are negligible. Excitingly, PTT mediated by silica core/gold shell “nanoshells” (also known as auroshells) has entered human clinical trials, with promising results reported [5–7]. Moreover, preclinical studies have shown that PTT can greatly enhance the efficacy of chemotherapy [8–11], radiotherapy [12–14] and immunotherapy [15, 16] when deployed in tandem, indicating PTT has great promise to improve patient care in the decades to come.

NPs used for PTT are typically administered IV and then accumulate within tumor tissue by exploiting the inherently leaky vasculature and poorly organized lymphatic system; this phenomenon is known as the enhanced permeability and retention (EPR) effect [17–19]. For this passive accumulation to work well, the administered NPs must safely navigate the blood stream where they are exposed to various serum and opsonin proteins that can cause their rapid clearance by tissue resident macrophages of the liver and spleen. The success of PTT relies upon the sufficient intratumoral accumulation of photoresponsive NPs to enable effective tumor heating upon irradiation [20, 21]. Numerous surface coatings have been employed to reduce NP opsonization and clearance and maximize tumor delivery, as elaborated in Sect. 1.2.

### Surface modification of NPs to maximize tumor delivery: the rationale for membrane coating strategies

Researchers have modified the surface of NPs with various moieties to achieve “passive” and/or “active” tumor targeting and minimize clearance by the mononuclear phagocytic system (MPS) [22, 23]. Passive targeting occurs when NPs accumulate in the tumor interstitial space by traveling through inter-endothelial cell gaps (the EPR effect) or by exhibiting transendothelial transport [24–26]. Active targeting occurs when NPs are functionalized with bioactive ligands, such as peptides, antibodies, aptamers, or proteins, that bind to specific surface receptors overexpressed on tumor cells and thereby improve NP retention at the target site [22, 23]. Traditionally, “stealth-coating” of NPs involves modification with bioinert polyethylene glycol (PEG) molecules, which provide steric stabilization and reduce opsonization by acting as a flexible polymer brush layer [27]. PEGylation increases NP blood circulation half-life, reduces MPS clearance, and increases tumor delivery versus unmodified NPs [23, 28]. However, PEGylation can cause an “anti-PEG” immunological response to trigger the accelerated blood clearance (ABC) phenomenon*,* wherein second doses of PEGylated NPs given several days after the initial injection are rapidly cleared from circulation [28–31]. Along with potential immunogenicity, PEGylation enables only passive tumor delivery, so alternative coatings are needed to increase NP specificity and accumulation in tumors [32–34].

To improve cancer cell-specific binding and tumor retention, NPs have been coated with receptor-specific ligands or cell-penetrating peptides [23, 35]. However, designing effective active targeting strategies is complex owing to the heterogeneous surface receptor expression on target cells and the difficulty producing NPs with the desired density and diversity of ligands [22, 23, 35]. Despite the promise of the concept, actively targeted NPs have demonstrated only modest improvements in tumor delivery over non-targeted NPs, in part because they still exhibit low circulation time, premature systemic clearance, and unwanted immune responses to surface ligands [35, 36]. Further improvements are required for active targeting methods to reach their full potential.

Independent of the type of surface modification, evidence has shown that only a limited amount (< 5%) of IV-administered NPs successfully reach tumors [24, 37]. This unsatisfactory delivery may be due to conventional ligand and PEG conjugation strategies not directly replicating the abundance, variety, and complexity of proteins found on naturally occurring cell membranes, which is critical to enhance target cell binding, or the inability of these strategies to avoid immune recognition and clearance by the MPS [23]. To overcome these limitations, researchers have started designing biomimetic NPs, wherein cytoplasmic membranes are extracted from desired cells in vitro and wrapped around NP exteriors to enhance their in vivo performance. Because membrane-wrapped NPs exhibit the highly complex combination of surface receptors, proteins, and phospholipids found on natural cell membranes, they can achieve effective biointerfacing. Zhang and colleagues were the first to synthesize biomimetic NPs by coating poly(lactic-co-glycolic acid) (PLGA) NPs with red blood cell (RBC) membranes [38]. These RBC-PLGA NPs exhibited significantly better stability and prolonged circulation in blood over PEG-coated and unwrapped controls [38]. This is believed to be due to the presence of CD47 “marker-of-self” proteins found on the RBC membranes that inhibit phagocytosis by immune cells. By coating NPs with other cell membranes, such as those derived from cancer cells, both immune evasion and active tumor targeting can be achieved by taking advantage of the ability of membrane-expressed proteins to facilitate cell–cell interactions [27, 39]. This is described in more detail in Sect. 3, where the benefits of different cells as membrane sources are summarized.

Owing to the improved biointerfacing of membrane-wrapped NPs, researchers are now exploring these biomimetic NPs for PTT with the goal of enhancing tumor delivery and therapeutic effects (Scheme 1C). This review summarizes the types of NP cores (Sect. 2, Table 1) and membranes (Sect. 3, Table 2) that have been used in PTT systems. Additionally, it discusses specific examples of membrane-wrapped NPs that have been utilized for PTT alone or in combination with other treatment modalities (Sect. 4). Lastly, it presents remaining challenges to be addressed and future directions for the field (Sect. 5).

**Table 1:** Different cores used in biomimetic PTT systems and their unique capabilities

Core type	Common irradiation wavelengths (nm)	Unique capabilities beyond PTT	References
Gold-based	785, 808, 850, 980, 1064	•Inherent PTT capacity can be tuned by changing geometric properties •Can be loaded with or conjugated to various molecules to provide imaging and/or drug delivery •Can enhance radiotherapy	[40–48]
Polymeric (loaded with organic NIR dyes)	Dependent on photo-activated cargo, includes: 765, 808, 1064	•Potential for co-loading with drugs or photosensitizers that enable chemotherapy or photodynamic therapy (PDT) along with PTT •Possibility to load with contrast agents that support many imaging methods (fluorescence, MRI, photoacoustic)	[49–53]
Prussian blue	808	•Additional molecules can be tethered to the particle surface •Potential for drug loading •Particles can have additional photo-responsive properties including PDT and photoacoustic imaging •Can support pH responsive release	[54–58]
Non-gold metal-based	808, 1064	•Many have natural PTT and imaging (MRI, photoacoustic, computed tomography) properties •Additional therapies include PDT, sonodynamic therapy, radiotherapy •Potential for drug and small molecule loading	[59–71]
Liposome	Dependent on photo-activated cargo, includes: 808, 1064	•Can alter irradiation wavelength depending on selected cargo •Additional molecules can be loaded to enable fluorescence imaging, PDT, or drug delivery	[72–74]
Nanosheets	808	•Drugs and other molecules can be adsorped or coordinated onto surfaces •Materials can be biodegradable	[75–78]
Carrier-free	Dependent on photo-activated cargo, includes: 680, 808	•No excess materials needed •Particles consist directly of drugs and other molecules to be delivered •Other components can be incorporated to provide PDT or imaging capabilities	[79–81]
Other materials	Naturally imbued or can depend on photo-activated cargo, includes: 808, 980	•Can have natural photothermal properties or be loaded with photo-responsive molecules •Semiconducting polymers can heat in response to light without having to load additional molecules •Can be loaded with diverse cargo providing immune stimulation, drug delivery, fluorescence imaging, or radiotherapy benefits •Some designs support pH responsiveness	[82–96]

**Table 2:** Different membrane types used in phototherapeutic NP systems, along with a summary of their characteristics

Membrane source(s)	NP components	Irradiation wavelength	Particle purpose (Beyond PTT)	References
MCF-7	PLGA, ICG	808	PAI, FI	[105]
4T1	Gold, DOX	808	Hyperthermia-activated drug delivery	[40]
4T1	Gold, mesoporous silica, R837	808	Immunostimulant, starvation therapy	[82]
4T1	Porous silicon, DOX, ICG	808	Drug delivery, FI	[83]
KB	Gold, PEG	980	Radiotherapy	[41]
A549	PLGA, perfluorocarbons (PFCs), ICG	765	FI, MRI, PAI	[49]
HepG2	Prussian blue, zinc glutamate, triphenylphosphine-conjugated lonidamine	808	Downregulated ATP production to disrupt heat shock proteins	[54]
MDA-MB-231	Gold, mesoporous silica, PTX, DOX	980	Drug delivery	[42]
B16-F10, 4T1, COS-7	Poly(caprolactone), pluronic copolymer F68, ICG	808	FI	[98]
C6	Proteolipids, ICG	808	FI	[72]
C6	Liposome, ICG	808	FI, PDT	[73]
HeLa	DOX, ICG	808	Drug delivery, FI	[79]
HeLa	Black-titanium, iridium	1064	PAI, sonodynamic therapy, luminescence	[59]
CT26	Bismuth	808	–	[60]
Platelet	Gold	808	–	[43]
Platelet	PLGA, IR780, DOX	808	Drug delivery	[51]
Platelet	Liposomes, IR 1048	1064	PAI	[74]
Platelet	Mesoporous silica, bismuth	808	Radiotherapy	[67]
MSC	Lipids, gold, iron oxide, DOX	808	Drug delivery, PAI	[68]
MSC	Black phosphorus, oxaliplatin (1,2-diaminocyclohexane) platinum (II)	808	Drug delivery	[77]
MSC	Polydopamine, SN38	808	Drug delivery	[86]
Macrophage	Gold	808	–	[44]
Macrophage	Iron oxide	808	–	[69]
Macrophage	DSPE-PEG, IR-792	808	FI	[52]
Macrophage	Bismuth selenide, quercetin	808	CT, drug delivery, heat shock protein inhibitor	[71]
Macrophage	Gold, ICG, triphenylphosphonium (TPP)	808, 1064	PDT, FI, PAI, Raman imaging	[45]
RBC	Iron oxide	808	MRI	[61]
RBC	Prussian blue, DOX	808	Drug delivery	[55]
RBC	Gold, poly(vinylpyrrolidone)	850	–	[46]
RBC	Prussian blue, Gamabufotalin (CS-6)	808	Drug delivery	[56]
RBC	Iron oxide	808	MRI	[62]
RBC	Gold	785	PAI	[47]
RBC	Melanin	808	PAI	[92]
RBC	Graphene oxide, ICG, DOX, DSPE-PEG	808	Drug delivery, FI	[75]
RBC	Semiconducting polymer, DSPE-PEG	808	PAI	[87]
RBC	Halloysite, FITC, ICG	808	FI	[89]
RBC	Poly(caprolactone), DPPC, Pluronic F68, PTX	808	Drug delivery	[95]
RBC	Black phosphorus	808	Immunotherapy	[78]
RBC	Gold, PTX, anti-EpCam	808	Drug delivery	[48]
RBC	Tungsten disulfide, PEG, DOX, ICG	808	Drug delivery, PDT, CT, FI	[63]
RBC	Prussian blue, J5, folic acid	808	Drug delivery	[57]
RBC	Zinc phthalocyanine, ICG	680	PDT, FI	[80]
RBC	Prussian blue, chlorin e6	808	PDT	[58]
RBC	10-Hydroxycamptothecin, ICG	808	Drug delivery, FI	[81]
RBC	Poly(caprolactone), PEG, IR780, DTX	808	FI, PDT, PAI, drug delivery	[53]
RBC	Graphene oxide, ICG, DOX, PEG, folic acid	808	Drug delivery, FI	[76]
RBC	Red blood cell content, copper sulfide, silica	980	Radiotherapy, oxygen delivery	[90]
Fibroblast	Poly(cyclopentadithiophene-alt-benzothiadiazole), PEG-b-PPG-b-PEG	808	PDT, PAI, FI	[96]
Myeloid derived suppressor cell	Iron oxide	808	MRI	[70]
CAR-T	Silica, IR780	808	FI	[91]
MCF-7 and RBC	Melanin	808	PAI	[88]
B16-F10 and RBC	DOX, copper sulfide	1064	Drug delivery	[64]
E. coli DH5α and B16-F10	Polydopamine	1064	Immunotherapy	[94]
4T1 and Dendritic Cell	Poly(benzobisthiadiazole-alt-thiophene), PEG-b-PPG-b-PEG, silicon 2,3-naphthalocyanine bis(trihexylsilyloxide)	1064	Immunotherapy, FI	[84]
Leukocyte and platelet	Silicon, IR780, DOX	808	PDT, drug delivery	[93]
ID8 and RBC	ICG, magnetic	808	Immunotherapy	[65]
Erythrocyte and platelet	Polypyrrol	808	–	[85]
WSU-HN6 and RBC	TPZ, IR780, H40-poly(ethylene glycol)	808	Hypoxia-activated drug delivery	[50]
CT26 and RAW 264.7	Triple-doped zinc gallogermanate, mesoporous silica, IR825, irinotecan	808	Drug delivery, luminescent imaging	[66]

*PAI* photoacoustic imaging; *FI* fluorescence imaging; *MRI* magnetic resonance imaging; *PDT* photodynamic therapy; *CT* computed tomography

## Types of photothermally active NP cores used in biomimetic systems

Various photoresponsive NPs have been wrapped with different kinds of cell membranes to enable PTT of solid-tumor cancers. This includes gold-based NPs (such as nanoshells, nanorods nanocages, nanostars, and nanodendrites) [40–48], polymeric NPs loaded with organic near-infrared (NIR) absorbing dyes (such as indocyanine green (ICG), IR780, IR820, and IR792) [49–53], Prussian blue NPs [54–58], non-gold metal-based NPs [59–71], liposomes [72–74], nanosheets (e.g., graphene-oxide, tungsten disulfide, and black phosphorous) [75–78], carrier-free systems made by co-assembling organic NIR dyes with other drugs [79–81], and other materials [82–96] (Table 1).

The intended application should guide the selection of a specific photosensitive NP core with the desired and necessary properties. For PTT as a standalone treatment, gold-based NPs are the leading cores due to their various benefits that include facile preparation, biocompatibility, high photothermal conversion efficiency, and ability to serve as contrast agents for optical imaging modalities [21, 40–48]. Additionally, by changing the structural dimensions of gold NPs, their plasmon resonance can be tuned to maximally absorb NIR light which is ideal for PTT. NIR light includes wavelengths ranging from 650 to 1350 nm and can safely penetrate a few centimeters into tissue because it has minimal absorbance by water and hemoglobin [97]. Most gold-based NPs absorb light within the first (650–850 nm) NIR window, but some have been designed to absorb light in the second (950–1350 nm) NIR window to enable maximal light absorption in deeper-tissue tumors [21]. Despite the great advantages of gold-based NPs, they are slowly excreted from the body owing to their non-biodegradable nature and they have low drug loading capacity compared to other materials [36]. Hence, researchers have also explored various degradable materials as the core of membrane-wrapped NPs for PTT [72–74, 79–81].

To enable PTT and contrast enhancement of tumors via magnetic resonance imaging (MRI), photoacoustic imaging, or computed tomography, possible NP cores include those based on gold or other metals that have inherent contrast-enhancing capabilities, as well as materials like polymers and liposomes loaded with NIR dyes or other contrast agents [44, 45, 47, 49–53, 59, 61–68, 70–74, 87, 95, 96, 98]. An advantage of polymer- or liposome-based NPs is that they may be co-loaded with drugs to enable combinatorial chemotherapy, imaging, and PTT (or simply dual chemo/PTT if the imaging agent is excluded) [50, 51, 53, 66, 84, 86, 95]. The appropriate material choice and NP design will depend in part on whether the cargo to be encapsulated is hydrophobic or hydrophilic. Importantly, wrapping cargo-loaded NPs with cell-derived membranes is advantageous in that it can slow drug release, preventing premature leakage throughout the body and enhancing tumor delivery to maximize therapeutic effects [34, 39, 99]. Several studies have shown that drug release from photothermally responsive membrane-wrapped NPs is both pH- and NIR light activation-dependent, allowing for high precision therapy [50, 64, 86]. Despite the good biocompatibility and biodegradability of organic liposomal and polymer-based NPs, they have inherent limitations compared to inorganic NPs including low photothermal conversion efficiency and poor photothermal stability under repeated laser irradiation [100]. Overall, the composition of the NP core is an important consideration for designing cell membrane-wrapped photothermal converters as it dictates their heating efficiency, stability, cargo-release kinetics, biocompatibility, and overall performance once the NP has been guided to the desired cancer cells by the membrane layer.

## Membrane types used to wrap phototherapeutic NPs

**Scheme 2:**
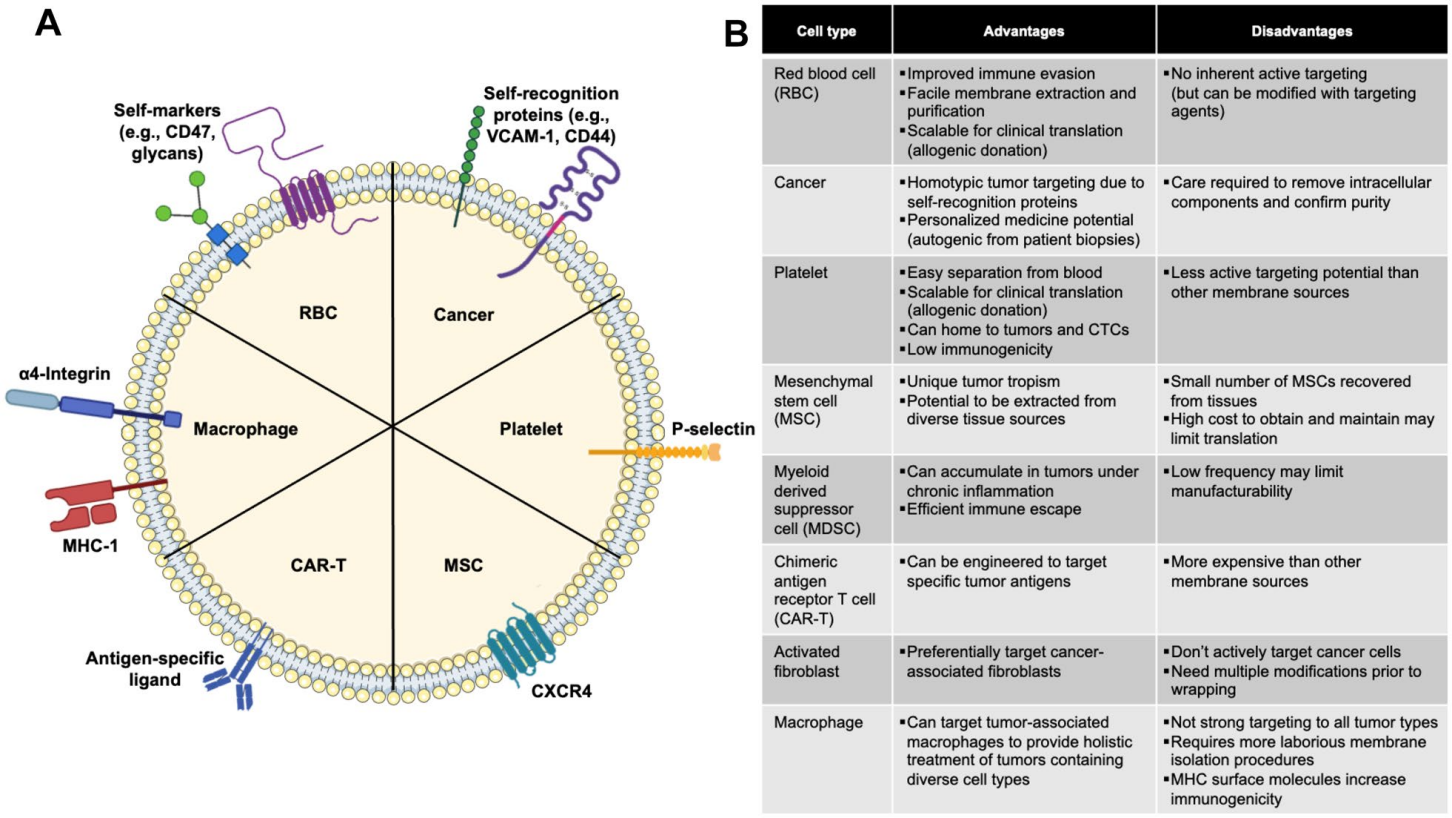
**A** Depiction of the composition of membrane-wrapped NPs, including representative proteins expressed on membranes from different cell sources, and **B** a summary of how these differences result in distinct advantages and disadvantages for various membrane-wrapped NPs. Portions of this figure were produced using Servier Medical Art templates (https://smart.servier.com). Servier Medical Art by Servier is licensed under a Creative Commons Attribution 4.0 Unported License. Portions of this figure were created with BioRender

Diverse cell types can be used as membrane sources to produce biomimetic NPs, with each membrane type offering unique features and capabilities that are derived from the specific proteins present in the bilayer structure (Scheme 2, Table 2). For example, RBC membrane coatings impart NPs with improved immune evasion owing to the presence of “self-markers” [53, 56, 63, 95]. Specifically, CD47, acidic sialyl moieties, and glycans are the key components enabling improved biointerfacing [101]. CD47 is a “don’t eat me” signal that prevents macrophage phagocytosis [101]. This prolongs blood circulation, allowing RBC membrane-wrapped NPs to passively accumulate in tumors in greater amounts than unwrapped NPs. This was demonstrated in the context of PTT where drug-loaded Prussian blue NPs accumulated in higher amounts in tumors in mice than unwrapped controls to effectively ablate tumor masses by NIR irradiation-induced PTT and chemotherapeutic drug release [55, 56]. Similar enhancement of PTT has been observed with other RBC membrane-wrapped NPs [48, 53, 57, 58, 63, 75, 76, 78, 81, 95]. Notably, self-markers are also present on membranes derived from other cell types, allowing them to achieve a degree of immune evasion.

One disadvantage of RBCs as a membrane source is that they cannot actively target surface receptors on tumor cells and hence support only passive accumulation. However, RBC membranes can be modified to add tumor targeting agents, such as the insertion of folate to support interaction with folate receptors overexpressed on cancer cells [63]. Although RBCs lack innate tumor targeting, their abundance in blood and their lack of a nucleus and complex organelles makes their membrane extraction and purification more facile and scalable for clinical translation [39]. Another advantage of RBCs as a clinically translatable membrane source is their allogeneic donation potential—RBCs can be acquired from donors to create biomimetic NPs that can be injected into patients with low risk of rejection, similar to the process for whole blood donation and transplants.

As an alternative to RBC membranes, cancer cell membranes are commonly applied as single membrane coatings because they offer both immune evasion and homotypic tumor targeting abilities. Homotypic tumor targeting is believed to be mediated by “self-recognition” proteins present on cancer cell membranes, as tumor cells strongly adhere to one another to form primary tumors and disseminated metastases [102, 103]. Due to their unique homologous adhesion properties, cancer cell membrane-wrapped NPs have been studied for diagnostics, therapeutics, and anticancer vaccine development, [104] as well as for PTT [40–42, 49, 54, 59, 60, 72, 73, 79, 82, 83, 98, 105]. Although cancer cell membrane-wrapped NPs could be synthesized using cancer cells directly obtained from patients via biopsies or surgery to create a personalized treatment, great care must to taken to confirm that all intracellular components have been removed to eliminate any carcinogenic potential [104]. In addition, some studies indicate the immune evasion of cancer cell membrane-wrapped NPs is limited compared to RBC or hybrid membrane wrapped NPs [65, 66]. These limitations must be weighed against the advantage of homotypic targeting when designing membrane-wrapped NPs for PTT.

Platelets are an intriguing third potential membrane source due to their easy separation from blood, allogeneic nature, and tumor homing capabilities [74]. Platelets can bind specifically to tumor cells through P-selectin anchoring to CD44 receptors on cancer cells [74]. Additionally, they have low immunogenicity because they express very few antigens on their plasma membrane [104]. These properties were shown to allow platelet membrane-wrapped NPs to specifically eliminate circulating tumor cells and inhibit cancer metastasis following PTT in an orthotopic murine breast tumor model [106]. Future research into platelet membrane-wrapped NPs for PTT is warranted given their desirable properties.

Like platelets, mesenchymal stem cells (MSCs) have promise as biomimetic NP coatings due to their unique migration and tumor tropism, which are mediated in part by surface receptors on their plasma membranes [77]. Source-related advantages of MSCs include their ease of isolation from in vitro cell culture and potential to be extracted from diverse tissues (i.e. bone marrow, adipose tissue, umbilical cord, or placenta) [77, 107]. Endogenous and exogenous MSCs migrate to specific tissues in response to chemokines and growth factors released at the target site. This is relevant to cancer therapy because MSCs use this homing effect to migrate to developing tumors. For example, tumors secrete stromal cell-derived factor-1 (SDF-1), which binds CXCR4 on MSCs, and this interaction can support the tumor accumulation of MSCs and MSC membrane-wrapped NPs [68, 108]. Despite MSCs’ tumor homing abilities, safety in allogeneic transplantation, and variety of tissue sources, the actual number of MSCs recovered from tissues is small and the high cost of obtaining and maintaining these MSCs may limit the clinical translation of MSC membrane-wrapped NPs for PTT compared to other cell membrane sources [104].

Macrophages are an attractive membrane source for biomimetic NPs because these circulating sentinels have the ability to distinguish, phagocytose, and eliminate foreign materials and malignant cells, including cancer and inflammatory cells [52, 66]. Although the active targeting of macrophages to tumors is not strong towards all tumor types, the presence of α4 integrin on macrophage membranes allows for binding to cancer cells that overexpress vascular cell adhesion molecule-1 [52, 109]. This α4 integrin/VCAM-1 binding was exploited to promote the accumulation of macrophage membrane-wrapped NPs in MDA-MB-231 primary tumors and lung metastases in mice in a recent study [71]. Beyond active targeting of cancer cells, macrophage membrane-wrapped NPs can target tumor-associated macrophages, allowing for more wholistic treatment of tumors containing diverse cell types [35, 71, 110]. Despite these advantages, macrophages require more laborious membrane isolation procedures that may reduce the membrane’s integrity and translocation of surface molecules [104]. Additionally, the expression of major histocompatibility complex (MHC) surface molecules may increase immunogenicity compared to the other membrane sources.

Several other cell types have shown promise as membrane sources for biomimetic phototherapeutic NPs. Myeloid derived suppressor cells (MDSCs) are promising membrane sources because they can accumulate in tumors in response to cytokines and chemokines secreted by tumor cells under chronic inflammation [70]. MDSC membrane-wrapped NPs thus exhibit efficient immune escape and tumor delivery [70]. Alternatively, activated fibroblast membranes can be coated onto NPs to preferentially target and photothermally ablate cancer-associated fibroblasts, which are the predominant population of tumor stromal cells in the tumor microenvironment and act as the key barrier to cancer therapies and diagnostics [96]. As the success of any therapy hinges on its ability to reach most of the tumor volume, using activated fibroblasts is an innovative way to target a larger cell subpopulation deep within and throughout the tumor stroma. Finally, membranes derived from chimeric antigen receptor T cells (CAR-T cells) that are designed to bind specific tumor-associated antigens have been wrapped around phototherapeutic NPs to endow them with tumor targeting abilities [91]. Overall, many membrane sources are available to wrap phototherapeutic NPs, each with advantages and disadvantages.

While membrane coatings are typically sourced from single cells, they can also be prepared from combinations of multiple cell types to create “hybrid” biomimetic NPs that can achieve increasingly complex tasks. The key advantage of hybrid membrane coating is that the desirable functions of each cell type can be maintained and utilized to overcome the limitations of the other. For example, the lack of tumor targeting of RBCs can be compensated by combining RBC membranes with cancer cell membranes that have homologous tumor targeting all while maintaining RBCs’ self-marker proteins that evade phagocytosis [50, 64, 88]. In the same sense, RBCs can be combined with platelets to add a tumor homing feature while maintaining long circulation [85]. Cancer cell membranes have also been combined with membranes derived from dendritic cells and macrophages to create biomimetic NPs capable of homologous targeting and enhanced immune escape [66, 84]. Overall, these combined features have increased the half-life of NPs in vivo and enriched tumor delivery compared to single membrane-wrapped NPs [33, 66]. For these strategies to be maximally effective, the appropriate ratio of membrane components must be determined experimentally. Wang et al. tested 4 different ratios of RBC and B16-F10 melanoma membranes to determine the optimal ratio that maximized blood circulation and tumor accumulation [64]. Researchers developing hybrid membrane-wrapped NPs for PTT should perform such rigorous studies to increase knowledge and enhance the potential for success. After synthesis, hybrid membranes should be characterized using SDS-PAGE, Western Blot, and/or immunofluorescence assays to ensure surface proteins from each source cell type are successfully transferred in the intended amounts onto the new biomimetic NPs. These confirmational characterization studies will help determine the relative amounts of different membrane components that endow certain biointerfacing properties onto hybrid membrane-wrapped NPs*.* By combining structural analysis with functional assays, researchers can begin to develop design rules for membrane-wrapped NPs that will ensure their successful implementation.

**Scheme 3:**
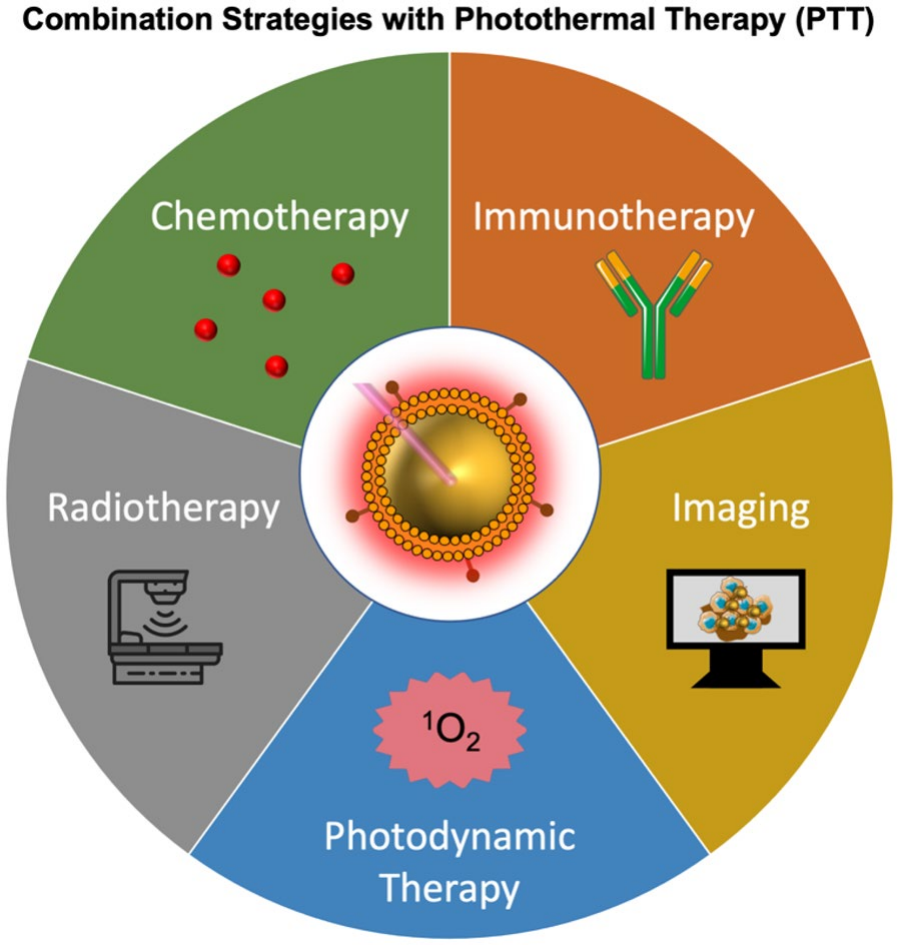
Membrane-wrapped NPs are being developed to enable PTT alone and in combination with other treatment or imaging modalities to ensure complete ablation of primary tumors and metastatic lesions and provide lasting anti-tumor effects that prevent recurrence. Portions of this figure were produced using Servier Medical Art templates (https://smart.servier.com). Servier Medical Art by Servier is licensed under a Creative Commons Attribution 4.0 Unported License

In summary, various cells can be used as membrane sources alone or in combination to produce membrane-wrapped NPs with the necessary immune evasion and/or tumor targeting abilities to support efficient and effective PTT. The most current studies including in vivo work in rodents have shown that membrane-wrapped NPs have improved circulation and tumor delivery compared to their unwrapped counterparts. However, since membrane-wrapped NPs are a relatively new technology, there has been limited to no extensive analysis of their pharmacokinetics or pharmacodynamics, which will be important for clinical translation. Future preclinical investigations should perform absorption, distribution, metabolism, and elimination (ADME) studies to collect pharmacokinetics information such as drug-clearance, bioavailability, exposure time, half-life, and distribution volume [111]. Moreover, researchers should evaluate the pharmacodynamics and efficacy of membrane-wrapped NPs in preclinical studies to identify the dosing regimen that induces maximum therapeutic effect. For a more detailed discussion and guide on how to evaluate pharmacokinetics and pharmacodynamics, readers can refer to a previous review that discusses the best practices for preclinical in vivo testing of cancer nanomedicines [111]. The following section describes specific examples of membrane-wrapped NPs used for PTT alone or in combination with other treatment modalities (Scheme 3).

## Membrane-wrapped NPs used for PTT in preclinical studies

### Membrane-wrapped NPs for PTT as a standalone therapy

PTT mediated by membrane-wrapped NPs is rapidly developing as a standalone therapy for solid-tumor cancers because it offers precise tumor ablation. Various kinds of NPs (e.g., nanoshells [44], nanorods [43], nanocages [46], and dye-loaded polymer NPs [91]) have been wrapped with different types of cell membranes (e.g., those derived from macrophages [44, 69], RBCs [46, 89], or cancer cells [60, 73]) to improve their intratumoral accumulation and photothermal effect. When evaluated in murine models, these membrane-wrapped NPs have generally exhibited prolonged circulation, improved tumor delivery, and better PTT effects than their unwrapped or PEG-coated counterparts [43, 44, 46, 60, 69, 85, 89, 91]. Below, a few specific examples are discussed to introduce readers to developments in this field.

As gold-based NPs are the leading PTT agents owing to their facile synthesis, tunable surface plasmon resonance (SPR), bioinertness, and high photothermal conversion efficiency, several researchers have explored the benefits of membrane-wrapping these NPs for PTT. For example, Xuan et al. wrapped mesoporous silica core-gold shell nanoshells with macrophage cell membranes and evaluated their impact against triple-negative breast cancer (TNBC), using unwrapped NPs as a comparative control [44]. The mesoporous silica was loaded with Cy7 to enable in vivo fluorescence imaging, and the silica core-gold shell thickness ratio was tuned such that the peak SPR was near 800 nm. Coating the NPs with macrophage membranes slightly red-shifted the SPR peak and did not hinder the heat conversion properties; when diluted to 1 mg/mL in aqueous solution the NPs’ temperature increased  ~ 30 °C within 5 min of irradiation with an 808 nm laser at 1 W/cm^2^. The anti-tumor effect of this platform was tested in male BALB/c nude mice bearing 100 mm^3^ subcutaneous 4T1 murine breast tumors. Twenty minutes after saline, unwrapped NPs, or wrapped NPs were administered IV, the tumors were irradiated with an 808 nm laser at 1 W/cm^2^ for 5 min. Tumor growth was monitored over 25 days, during which only the mice treated with membrane-wrapped NPs and the laser experienced nearly complete tumor regression; all other treatment groups exhibited tumor growth. A biodistribution study showed that the macrophage membrane-wrapped NPs had longer circulation and  ~ 4.6X greater tumor accumulation than their unwrapped counterparts (7.48% injected dose per g (ID/g) in tumors for membrane-wrapped NPs versus 1.61% ID/g for unwrapped NPs), which likely contributed to their improved PTT effect [44].

In a similar study, Piao et al. functionalized photoresponsive gold nanocages (AuNCs) with poly(vinylpyrrolidone) (PVP, a biopolymer that is a potential alternative to PEG) and wrapped these PVP-AuNCs with RBC membranes [46]. Wrapping slightly reduced and red-shifted the peak SPR wavelength from ∼810 to ∼817 nm. While RBC-AuNCs exhibited slightly less heating upon 850 nm irradiation than PVP-AuNCs, the overall temperature increase was still sufficient for PTT, as the temperature rose by  > 20 °C within 2 min of irradiation. The anti-tumor effect of this platform was tested in female BALB/c nude mice with subcutaneous 4T1 tumors. Pharmacokinetic and biodistribution studies showed that the RBC-AuNCs had a circulation half-life of 9.5 h, compared to just 1 h for PVP-AuNCs. Further, at 24- and 48-h post-injection, the intratumoral accumulation of RBC-AuNCs was 8.34% and 7.77% ID/g, respectively, compared to 4.37% and 4.46% ID/g for PVP-AuNCs. In a follow-up efficacy study, tumor-bearing mice were irradiated with an 850 nm laser (1 W/cm^2^, 10 min) 2 days after IV administration of saline, PVP-AuNCs, or RBC-AuNCs. PTT mediated by both the PVP-AuNCs and RBC-AuNCs reduced tumor volume, but only RBC-AuNC-mediated PTT resulted in 100% survival over a span of 45 days. Interestingly, the authors found contradicting results when testing this treatment in vitro and in vivo. Membrane-wrapping reduced NP cellular uptake and PTT efficacy in vitro, but improved PTT efficacy in vivo, likely due to the improved blood circulation and tumor accumulation.

The previous examples demonstrate the benefit of wrapping singular NPs with cell-derived membranes, but an alternative approach is to load entire cells with multiple NPs to mediate PTT. To compare these approaches, Rao et al. loaded bovine serum albumin-conjugated gold nanorods (AuNRs), which have a longitudinal SPR peak at  ~ 808 nm, inside whole platelets and compared them against AuNRs wrapped with platelet cell membranes [43]. In vivo, that platelet membrane-wrapped AuNRs (PLT-M-AuNRs) had similar, although slightly reduced, photothermal efficacy as AuNRs loaded within intact platelets (PLT-AuNRs). Specifically, PLT-AuNRs had longer blood circulation compared to PLT-M-AuNRs when administered IV to ICR mice. Additionally, the PLT-AuNRs exhibited improved tumor delivery when IV administered to mice bearing CAL27 head and neck squamous cell carcinoma tumor xenografts in the oral cavity. Accordingly, after the mice were irradiated with an 808 nm laser 24 h post-NP injection, tumor growth was slowest over the following 15 days in mice that received PLT-AuNRs plus laser, though the PLT-M-AuNRs plus laser also decreased tumor growth compared to AuNR plus laser. This study demonstrates that both membrane-wrapping of singular NPs and loading NPs within intact cells are promising approaches to enhance the efficacy of PTT versus traditional PTT mediated by non-biomimetic NPs.

Beyond gold-based NPs, other materials have been used to enable PTT including polymer-based NPs [85], mesoporous silica loaded with IR780 [91], liposomes loaded with ICG [73], metal bismuth NPs [60], inorganic magnetic iron oxide NPs [69], and inorganic halloysite nanotubes loaded with ICG [89]. The ability to wrap such materials with cell-derived membranes for enhanced PTT has shown great promise. For instance, Liu et al. wrapped polypyrrol NPs with RBC-platelet hybrid membranes to enable tumor-targeted PTT [85]. Polypyrrol is an organic photothermal polymer with good biocompatibility, high photothermal conversion efficiency, and excellent photostability under repeated irradiation. The hybrid membrane wrapping endowed the NPs with a dual tumor-targeting ability as the RBC membrane components increased immune escape to support passive tumor accumulation prior to laser irradiation, while the platelet membrane components allowed additional NPs to be recruited to the injured tumor site by damage induced during the initial PTT. The anti-tumor effect of this platform was evaluated in male nude mice with subcutaneous HCT116 human colon cancer tumors. Two hours after NP injection via the tail vein, the tumors were irradiated (808 nm laser, 1 W/cm^2^, 3 min), which increased the temperature to  ~ 50 °C and caused tumor vessel damage/inflammation that would recruit more NPs to the tumor. Four hours later, the tumors were irradiated again (808 nm laser, 1.5 W/cm^2^, 5 min), increasing the tumor temperature to 58.1 °C. This treatment method significantly decreased tumor volume such that tumors were nearly gone within 2 days of treatment and remained absent for 14 days of monitoring after treatment. While tumor volume initially decreased in mice treated with NPs coated with only RBC membranes or only platelet membranes, the tumors regrew in these groups over the 14-day study period, demonstrating the advantage of the hybrid membrane coating approach. This study also evaluated the appropriate ratio of RBC to platelet membranes by examining the pharmacokinetics of NPs coated with RBC and platelet membrane proteins at ratios of 1:1, 1:2, or 2:1. Polypyrrol NPs coated with a membrane protein ratio of 2 RBC:1 platelet exhibited the longest circulation time, and thus this formulation was used in the therapeutic study described above. The findings of this work show that hybrid membrane-wrapping strategies can be usefully employed to enhance PTT.

Beyond hybrid membrane wrapping, another approach to maximize tumor delivery of phototherapeutic NPs is engineering the source cell membrane to express tumor-targeting moieties. In an intriguing approach, Ma et al. wrapped IR780-loaded mesoporous silica NPs with CAR-T cell membranes to thermally ablate hepatocellular carcinoma tumors [91]. Mesoporous silica NPs were used as carriers for the NIR dye due to their tunable pore size, excellent biocompatibility, and high drug loading capacity. The authors genetically modified CAR-T cells via lentivirus transfection to enable targeting of glypican-3 (GPC3) proteoglycans that are overexpressed by GPC3 + hepatocellular carcinoma cells. Wrapping the NPs with the CAR-T cell membranes improved their stability, enhanced their tumor targeting, and slowed the release of IR780 without impairing its photothermal conversion. The anti-tumor effect of these NPs was tested in male BALB/c-nu mice with subcutaneous Huh-7 human liver cancer tumors. One day following NP injection, the tumors were irradiated with an 808 nm laser at 0.6 W/cm^2^ for 5 min. The mice were injected and irradiated every 3 days for 19 days. Tumor volume decreased significantly in mice treated with CAR-T cell membrane-wrapped IR780-loaded NPs and laser, with lesser tumor regression observed in mice that received either free IR780 or IR780-loaded unwrapped NPs in combination with laser irradiation. PTT mediated by CAR-T membrane-wrapped NPs was also more effective than PTT mediated by T cell membrane-coated IR780-loaded NPs, demonstrating the advantage of the GPC3 targeting. These findings indicate that CAR T-cell membrane-wrapped NPs have promise as PTT mediators, but further testing is needed to verify the safety and efficacy of such treatments before clinical use.

As an alternative to hybrid membranes or engineered CAR-T membranes, cancer cell membranes can be used to improve the tumor delivery of photothermal NPs via homotypic targeting, as shown in the work of Xu et al. [73]. They developed ICG-loaded liposomes wrapped with C6 glioma cell membranes, which had peak absorption at  ~ 800 nm, close to that of the free dye at 780 nm, and which also exhibited prolonged blood circulation time and improved accumulation in subcutaneous C6 glioma tumors in BALB/c nude mice. The impact of PTT was examined by irradiating 60 mm^3^ tumors 1-h post-IV NP administration with an 808 nm laser at 1 W/cm^2^ for 5 min. Within 18 days, the primary tumor was completely eradicated in mice treated with the C6 membrane-wrapped liposomes, with no tumor relapse or formation of lung metastases observed. By comparison, neither free ICG nor unwrapped ICG-loaded liposomes completely eradicated tumors following irradiation, and some lung metastases were identified in these mice post-mortem. Given that metastasis is a leading cause of cancer death, future work should study the mechanism by which the homotypic PTT inhibited lung metastasis and increased antitumor immune response.

Other examples of membrane-wrapped NPs explored for PTT alone include colon cancer cell membrane-wrapped bismuth NPs (which support homotypic tumor targeting) [60], and macrophage membrane-wrapped magnetic iron oxide NPs (which support tumor delivery through both cell adhesion molecules present on the macrophage membranes and through NP attraction to an externally applied magnet) [69]. Another example includes halloysite nanotubes loaded with ICG which were coated with antibody-modified RBC membranes to target epithelial cell adhesion molecule (EpCAM) receptors that are overexpressed on breast cancer cells [89]. These examples and those discussed above demonstrate that a plethora of membrane-wrapping strategies can be utilized to enhance the success of PTT.

**Scheme 4:**
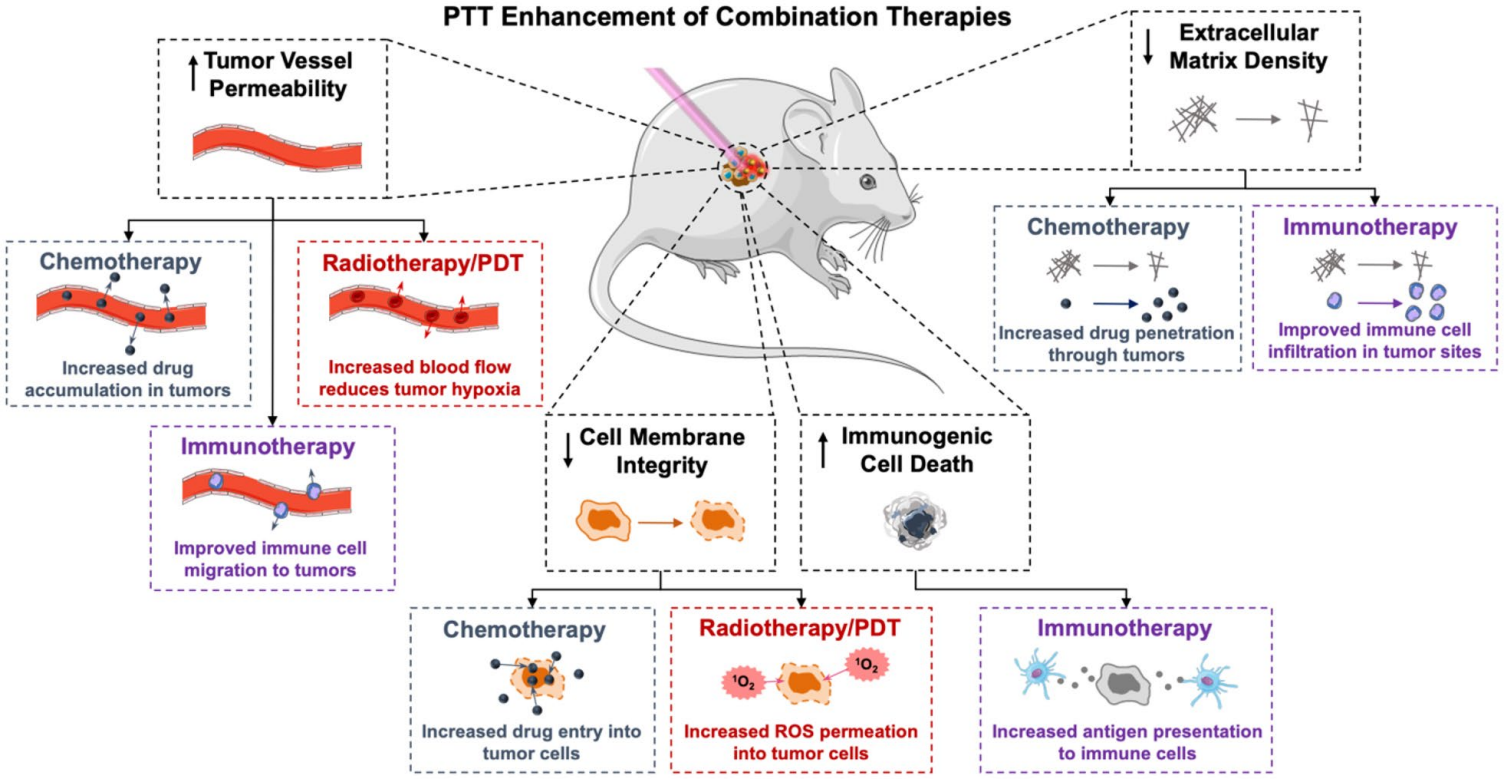
PTT has numerous impacts on the tumor microenvironment that can potentiate the effect of concurrently applied chemotherapy, radiotherapy, photodynamic therapy (PDT), or immunotherapy. Portions of this figure were produced using Servier Medical Art templates (https://smart.servier.com). Servier Medical Art by Servier is licensed under a Creative Commons Attribution 4.0 Unported License

Across studies, membrane-wrapped NPs have outperformed their unwrapped counterparts, supporting continued development of membrane-wrapped NPs for PTT. Despite the success of these platforms to thermally ablate solid-tumor cancers under NIR irradiation, PTT as a standalone therapy has some limitations in the long term. For example, PTT can be ineffective if it fails to kill cancer cells in the primary tumor that are outside the irradiation region or if it fails to eliminate metastatic disease. To solve these issues, membrane-wrapped NPs have been developed that can both mediate PTT and provide contrast for various imaging modalities to ensure complete and precise ablation of the entire tumor [45, 49, 53, 54, 59, 61–64, 66, 70–72, 74, 87, 88, 96, 98, 105]. PTT is also being combined with other treatments like chemotherapy, photodynamic therapy, immunotherapy, and radiotherapy to enable a synergistic long-lasting anti-tumor effect (Scheme 3). Some of the mechanisms by which PTT enhances other therapies are depicted in Scheme 4. These advances in multimodality therapy mediated by photoresponsive membrane-wrapped NPs are described in the following sections.

### Dual PTT and chemotherapy faciliated by membrane-wrapped NPs

**Scheme 5.**
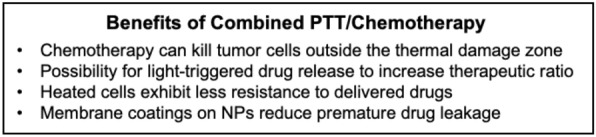
Summary of some of the benefits of combined PTT and chemotherapy

PTT has substantial promise in combination with chemotherapy as each treatment can overcome the limitations of the other (Scheme 5). The efficacy of chemotherapy is compromised by tumor heterogeneity, drug resistance, lack of specificity, and systemic toxicity. Conversely, PTT alone does not kill cancer cells outside the irradiation region, leaving the risk for remaining cancer cells to metastasize or cause recurrence. By combining chemotherapy with PTT using biomimetic NPs, both thermal cell ablation and hyperthermia triggered drug release inside tumors can be achieved. This allows lower doses of drugs to achieve satisfactory anti-tumor effects, minimizing dose-related side effects [112]. Several studies have demonstrated that combined PTT/chemotherapy mediated by biomimetic NPs is extremely effective and that the membrane layer prevents premature drug release, improves tumor delivery, and does not affect photothermal conversion efficiency [42, 51, 56, 81, 83, 86]. Additionally, some dual PTT/chemotherapy systems have demonstrated the ability to hinder both primary tumors and metastases in murine cancer models [40, 83, 95]. This section summarizes several examples from literature, with subsections divided by the type of material utilized as the NP core.

#### Biomimetic polymer NPs for dual PTT and chemotherapy

Several polymer-based biomimetic NPs have been explored for dual PTT and chemotherapy, including those derived from polydopamine (PDA) [86], hyperbranched PEG [50], poly(caprolactone) (PCL) [95], PLGA [51], and PCL-PEG-PCL triblock copolymers [53]. PDA is a bio-inspired polymer that exhibits high photothermal performance and excellent biocompatibility, and it can also be loaded with chemotherapeutics for hyperthermia-triggered drug release. Zhang et al. harnessed these abilities to prepare PDA NPs loaded with 7-ethyl-10-hydroxycamptothecin (SN38), a hydrophobic irinotecan analog, and wrapped with umbilical-cord mesenchymal stem cell (MSC) membranes [86]. The MSC membrane layer reduced macrophage interactions of the PDA NPs in vitro, prolonged circulation time in vivo*,* and approximately doubled the amount of NPs delivered to subcutaneous MG63 human malignant bone cancer xenografts in female BALB/c mice following IV administration. In vitro studies showed that SN38 release from the membrane-wrapped NPs was both pH- and temperature-dependent, allowing for enhanced drug release in the tumor microenvironment both due to its acidic nature and due to NIR irradiation. Efficacy studies showed that tumor volume decreased by 60% over a two-week period post-treatment in mice exposed to membrane-wrapped SN38-PDA NPs whose tumors were irradiated with an 808 nm laser at 0.6 W/cm^2^ for 5 min, which was statistically different from the 47% reduction in tumor volume observed without irradiation. This indicates that dual PTT/chemotherapy mediated by membrane-wrapped NPs is advantageous versus chemotherapy alone for malignant bone tumors.

In a similar approach, Chen et al. developed hyperbranched PEG (H40-PEG) NPs that were co-loaded with the photothermal dye IR780 and tirapazamine (TPZ), a drug that is activated in hypoxic environments [50]. These NPs were wrapped in a hybrid manner using membranes derived from head and neck squamous cell carcinoma cells (which support homotypic targeting) and RBCs (whose CD47 markers provide immune escape). The membranes were further functionalized with aspartic acid octopeptides (Asp8) that have a high affinity to hydroxyapatite to enable the NPs to target tumors within bones [50]. The membrane-wrapped NPs had a drug loading efficiency of 52% for TPZ and 60.90% for IR780, and the release of TPZ was both pH and irradiation/temperature dependent. Notably, the IR780 allowed not only the production of heat, but also the generation of singlet oxygen, which increased tumor hypoxia to activate the prodrug TPZ. This clever approach proved very effective when evaluated in female BALB/c nude mice bearing WSU-HN6 head and neck squamous cell carcinoma tumors in the right mandible. Ten days after tumor inoculation, mice received an IV injection of NPs or controls, followed by irradiation of the tumor (808 nm, 1 W/cm^2^, 5 min) on the subsequent two days. After a 1-day rest period, the treatment cycle of NP injection and 2 days of irradiation was repeated. This yielded excellent tumor growth inhibition, as the average weight of tumors excised on day 16 post-treatment initiation in the dual PTT/chemo group was three-fold lighter than that of tumors in the saline control group. A chemotherapy only group (i.e., Asp8-modified membrane-wrapped NPs without irradiation) was not included as a comparison, but the dual PTT/chemo was more effective than PTT alone mediated by bare H40-PEG-IR780 NPs. It was also more effective than dual PTT/chemo mediated by membrane-wrapped NPs without the Asp8 modification, although the difference in efficacy was not statistically significant. Importantly, the dual treatment approach did not destroy the jaw bones, demonstrating the precise nature of this therapy.

While the above examples demonstrate the potential of dual PTT/chemo against primary tumors, others have shown the potential of this approach to manage metastatic disease. For example, Su et al. developed PCL NPs loaded with paclitaxel (PTX) that were functionalized with 1,2-dipalmitoyl-sn-glycero-3-phosphocholine (DPPC), a thermally responsive material that would undergo phase transition upon NP heating to allow drug release [95]. The DPPC-modified NPs were wrapped with RBC membranes that were embedded with the NIR dye DiR to enable PTT. The resulting biomimetic NPs had encapsulation efficiency of 98.4% and 96.8% for DiR and PTX, respectively. The antitumor and antimetastatic effect of this platform was tested in female BALB/c mice bearing orthotopic 4T1 breast tumors that spontaneously metastasize to the lungs. Four hours after NP injection, the primary tumors were irradiated (808 nm laser, 3 W/cm^2^, 5 min), increasing the tumor temperature to 54.6 °C. This treatment was repeated 7 times at a 2-day time interval, which significantly suppressed primary tumor growth and resulted in a remarkably low metastatic rate. While saline-treated mice had an average of 57.6 metastatic nodules in the lung at the end of the study, the dual PTT/chemo group had only 1 metastatic nodule in one lung, indicating an astounding antitumor and antimetastatic effect. This treatment was also more effective against primary tumors and metastasis than PTT or chemotherapy mediated by membrane-wrapped NPs alone, demonstrating the benefits of combining PTT and chemotherapy in a single platform. These observations agree with the findings of Pei et al. who showed that platelet membrane-wrapped PLGA NPs carrying both doxorubicin (DOX) and IR780 could completely ablate subcutaneous 4T1 tumors in BALB/c mice without evidence of tumor recurrence in the 18 days post-treatment study period [51]. Overall, the studies noted here demonstrate the vast potential of biomimetic polymer NPs as mediators of dual PTT/chemotherapy. A final advantage of polymer-based NPs for dual PTT/chemo is that, depending on the specific design, they may also be loaded with contrast agents to support tumor imaging [53]. As image-guided therapy can improve disease elimination, this is an attractive feature of biomimetic polymer NPs.

#### Two-dimensional nanosheets as biomimetic platforms for dual PTT/chemotherapy

Two-dimensional (2D) nanosheets have also been explored for combined PTT/chemotherapy. Compared to spherical NPs, 2D nanosheets offer a large surface-to-volume ratio that provides high drug loading capacity. One example of a membrane-wrapped nanosheet utilized for dual PTT/chemotherapy was reported by Li et al. who coated 2D graphene oxide nanosheets that had ICG and DOX adsorbed to their surface with RBC membranes functionalized with folic acid (FA) to enable targeting to HeLa tumors that overexpress folate receptors [75]. The anti-tumor effect of this nanosheet design was tested in nude mice bearing subcutaneous HeLa cervical cancer tumors. The mice were IV injected with the modified nanosheets once daily for 2 days, and 48 h after the second treatment the tumors were irradiated (808 nm laser, 2 W/cm^2^, 5 min). The tumors in mice that received folate-modified, RBC membrane-wrapped nanosheets in combination with laser were totally absent 14 days post treatment, and the reduction was greater than that observed for dual PTT/chemo mediated by non-folate modified nanosheets or chemotherapy only mediated by either the full formulation without laser exposure or the non-folate modified system without laser exposure. In sum, this paper showed a novel approach of wrapping 2D graphene oxide with cell membranes for dual PTT/chemotherapy, supporting continued development of similar systems.

Other 2D nanomaterials that have been explored for dual PTT/chemotherapy include tungsten disulfide (which was modified with PEG, co-loaded with ICG and DOX, and wrapped with FA-modified RBC membranes) [63] and black phosphorus nanosheets (that were coordinated with the active species of oxaliplatin (1,2-diaminocyclohexane) platinum (II) (DACHPt) as the chemotherapeutic and coated with MSC membranes) [77]. The tungsten disulfide (WS_2_) system had a circulation half-life of 5.5 h when injected in healthy Balb/c nude mice, compared to just 1.5 h for unwrapped controls [63]. This system, which enabled simultaneous PTT, chemotherapy, and photodynamic therapy (PDT) owing to the inclusion of ICG and DOX, was further evaluated in female BALB/c mice with subcutaneous HeLa tumors. NPs were IV injected at equivalent concentrations of ICG (5 mg/kg) and DOX (2 mg/kg), and 24-h post injection the tumors were irradiated for 5 min (808 nm laser, 1 w/cm^2^). The membrane-wrapped WS_2_ NPs effectively increased tumor temperatures from 30 to 54.9 °C, resulting in a 95.4% tumor inhibition rate at the end of 8 treatment cycles [63]. By comparison, wrapped NPs without laser excitation inhibited tumor growth by 72.4% due to DOX delivery (i.e., chemo alone), The FA-modified system did not outperform the non-modified system in this study, which may indicate that the RBC membrane alone is sufficient to enrich nanosheet accumulation in the tumor.

The black phosphorous system mentioned above also performed extremely well in vitro, but remains to be tested in animal models [77]. Compared with graphene oxide, black phosphorus nanosheets have a much larger surface-to-volume ratio due to their puckered lattice configuration, making these materials a potentially greater drug carrier. Additionally, black phosphorous is biocompatible in vivo as it degrades into nontoxic phosphorus compounds. While black phosphorous is an inherently excellent photosensitizer for NIR-mediated PTT, it degrades in vivo in the blood. Coating this material with MSC membranes should not only provide stability to support in vivo circulation, but also improve tumor targeting owing to MSCs’ unique tumor tropism. This therapeutic platform was tested in vitro and exhibited improved dispersion in media and enhanced uptake by A549 cancer cells, resulting in successful dual PTT/chemotherapy. Future studies are needed to validate this system in vivo*,* but the results obtained to date are promising [77]. Overall, the studies noted here indicate that 2D nanomaterials wrapped with biological membranes have great promise as tools for multimodal cancer therapy.

#### Membrane-wrapped Prussian blue NPs for dual PTT/chemotherapy

Prussian blue NPs have also been wrapped with cell membranes and combined with drugs for dual PTT/chemotherapy. Prussian blue NPs are FDA-approved photosensitizers that offer excellent photothermal conversion efficiency in the NIR region. They are metal organic frameworks (MOFs) that offer large surface area, high volume, and tunable pore size, making them great drug carriers for NIR-triggered drug release. The first demonstration of membrane-wrapped Prussian blue NPs for dual PTT/chemo was reported by Chen et al. who synthesized RBC membrane-coated hollow mesoporous Prussian blue NPs loaded with DOX and evaluated them in male BALB/c mice bearing subcutaneous 4T1 tumors [55]. Mice received IV injection of the RBC membrane-wrapped, DOX-loaded Prussian blue NPs or various controls and 24 h later their tumors were irradiated (808 nm laser, 1 W/cm^2^, 5 min). PTT mediated by the biomimetic drug-loaded Prussian blue NPs increased tumor temperature up to 60.6 °C, resulting in a tumor growth inhibition rate of 98.3% over a 15-day period. Without irradiation the biomimetic drug-loaded NPs yielded growth inhibition of 43.9%, demonstrating that the added PTT significantly increased tumor response versus chemotherapy alone.

These exciting results opened the door for other groups to investigate membrane-wrapped Prussian blue NPs for combined PTT/chemotherapy. In one study, Prussian blue NPs were loaded with gamabutolin, a derivative of the traditional Chinese medicine Chansu, and wrapped with RBC membranes functionalized with hyaluronic acid (HA) for synergistic PTT/chemo of subcutaneous MDA-MB-231 breast cancer tumors in female BALB/c mice [56]. This system yielded a 93.4% tumor inhibition rate, corroborating that dual PTT/chemo mediated by drug-loaded Prussian blue NPs is extremely potent against breast cancer. This approach also appears to be effective against cervical cancer, as Daniyal et al. loaded cubic Prussian blue NPs with J5, a plant-based chemotherapeutic that is commonly used in Chinese folk medicine, and wrapped these NPs with FA-modified RBC membranes to enable combined PTT/chemo of HeLa tumors in female BALB/c mice [57]. NPs were injected on alternate days and 24 h after each injection the tumors were irradiated (808 nm laser, 1 W/cm^2^, 5 min), for a total of 10 treatment cycles. The resulting tumor inhibition rate was 94%, again demonstrating that dual PTT/chemo mediated by Prussian blue NPs is a highly effective cancer treatment.

Finally, Wang et al. loaded Prussian blue NPs with the anticancer drug lonidamine (which inhibits production of intracellular ATP to disrupt heat shock proteins that ordinarily protect cells from thermal stress) and wrapped these NPs with HepG2 hepatocellular carcinoma cancer cell membranes to facilitate treatment of subcutaneous HepG2 tumors in nude mice [54]. Twelve hours post-IV administration, NPs wrapped with HepG2 membranes exhibited tumor accumulation of 17.2 ± 2.1% ID/g (percent injected dose per gram) compared to only 9.4 ± 1.5% ID/g for unwrapped NPs. Upon 808 nm laser irradiation (1 W/cm^2^, 5 min), tumors of mice treated with HepG2 membrane-wrapped, drug-loaded Prussian Blue NPs heated to 44.9 ± 1.7 °C, resulting in a tumor inhibition rate of 89.2% over the course of 16-days post-treatment. Western blot and immunofluorescence confirmed that the released lonidamine reduced HSP70 and HSP90 expression levels, thereby enhancing the efficacy of mild PTT mediated by the NPs. Overall, the studies discussed here indicate biomimetic Prussian blue NPs have great potential as mediators of dual PTT/chemotherapy.

#### Membrane-wrapped gold NPs for photo-chemotherapy

As classical PTT agents, gold-based NPs have also been wrapped with cell membranes for synergistic PTT/chemotherapy to ablate solid tumors. Some gold NPs (such as nanocages) allow for drug loading within their interior, while others (like nanorods) require drugs to be tethered to their surface. Zhang et al. coated nanorods with a mesoporous silica shell to which a PTX prodrug was conjugated and DOX loaded. These dual drug-loaded nanorods were wrapped with MDA-MB-231 TNBC cell membranes to provide homotypic targeting to TNBC tumors [42]. Through this design, the co-loading of hydrophilic (DOX) and hydrophobic (PTX) drugs within one nanocarrier was possible to circumvent cancer drug resistance. Encapsulation with a mesoporous silica shell, co-loading of two drugs, and membrane wrapping did not impact the photothermal conversion efficiency of the nanorods; their temperature increased from 28 to 67.5 °C within 5 min of irradiation with a 980 nm laser at 1 W/cm^2^. The anti-tumor effect of this treatment was tested in female nude mice bearing subcutaneous 4T1 tumors. The mice were injected with NPs every 3 days and their tumors were irradiated the day after each NP injection (980 nm light, 1 W/cm^2^, 5 min). Tumor growth was significantly suppressed by the drug-loaded membrane-wrapped NPs compared to freely administered DOX + PTX. Even though the laser-activated membrane-wrapped NPs carrying the drugs achieved improved on-site drug release and anti-tumor effect in vivo, they did not exhibit a better anti-tumor effect than those without laser irradiation. The authors attributed this observation to the potential weak skin penetration of the applied light.

Compared to nanorods, nanocages offer the advantage of allowing drug loading within their interior because they have porous walls. Adding a membrane coating around drug-loaded nanocages prevents premature drug leakage*,* while also improving circulation and tumor delivery. In the work of Sun et al. gold nanocages were loaded with DOX and wrapped with 4T1 cell membranes, and this system was administered to female nude mice with orthotopic 4T1 tumors [40]. Primary tumors were irradiated (808 nm laser, 2.5 W/cm^2^, 5 min) every 3 days for four times after NP injection. Primary tumor volume was suppressed by 98.9% and lung metastases were dramatically decreased by 98.5%, demonstrating that biomimetic drug-loaded nanocages have great potential as anti-tumor and anti-metastasis agents. The impact of drug-loaded membrane-wrapped nanocages against 4T1 tumors in mice was also shown by Zhu et al. who loaded nanocages with PTX and wrapped the NPs with RBC membranes that were modified with anti-EPCAM (epithelial cell adhesion molecule) antibodies to allow the NPs to actively target EpCAM transmembrane proteins that are overexpressed in breast cancer [48]. Like the prior report, this study showed that dual PTT/chemotherapy mediated by drug-loaded membrane-wrapped nanocages is highly effective in murine tumor models. These studies affirm that combining the photothermal properties of gold nanocages with chemotherapy can achieve significant anti-tumor effects.

#### Alternative materials for dual PTT/chemotherapy

Beyond the systems noted in Sects. 4.2.1 through 4.2.4, many other materials have been explored for combined PTT/chemotherapy, including hollow copper sulfide (which was loaded with DOX and wrapped with hybrid RBC/B16-F10 melanoma cell membranes) [64] and ZGGO (Zn_1.25_Ga_1.5_Ge_0.25_O_4_:Cr^3+^,Yb^3+^,Er^3+^) persistent luminescence NPs (that were coated with a mesoporous silica shell co-loaded with IR825 and irinotecan and camouflaged with hybrid macrophage/colorectal cancer cell membranes) [66]. The biomimetic copper sulfide system was the first to fuse RBC and cancer cell membranes for NP functionalization and was evaluated in female BALB/c mice bearing subcutaneous B16-F10 melanoma tumors. Following NP delivery and tumor irradiation (1064 nm laser, 1 W/cm^2^, 5 min), the tumor temperatures reached 51.2 °C, resulting in 100% tumor growth inhibition [64]. The persistent luminescence NP system was advantageous in that it supported real-time imaging of the NPs in vivo along with enabling dual PTT/chemotherapy [66]. This platform was tested in BALB/c mice harboring subcutaneous CT26 colon cancer tumors, and effectively increased tumor temperatures by 22 °C upon 5 min of irradiation, resulting in a remarkable decrease in tumor growth compared to other treatments. Taken together, these studies indicate that non-traditional NPs that are wrapped with cell membranes have promise as tools for dual PTT/chemotherapy of solid-tumor cancers.

Carrier-free co-assemblies of NIR dyes and chemotherapy drugs have also been wrapped with biological membranes to provide combined PTT/chemotherapy. Zhang et al. co-assembled ICG and DOX to produce NPs that were wrapped with cervical cancer cell membranes [79], while Ye et al. developed a carrier-free assembly of ICG and 10-hydroxycamptothecin (10-HCPT), which was wrapped with RBC membranes [81]. Such carrier-free assemblies provide high drug payloads and may be safer than carriers comprised of materials that are foreign to the body, but they suffer from poor stability and pre-mature drug release. Fortunately, the presence of a membrane layer can overcome these limitations. Both membrane-wrapped carrier-free assemblies were evaluated in male BALB/c nude mice bearing subcutaneous HeLa tumors [79, 81]. The ICG/DOX system increased tumor temperatures from 37 to 52.4 °C within 5 min of irradiation (808 nm laser, 3 W/cm^2^), shrinking the tumor volume remarkably to 0.2-fold its original volume at the end of 2 treatment cycles [79]. Likewise, the ICG/10-HCPT system increased tumor temperature to 56 °C after 8 min of irradiation (808 nm laser, 1 W/cm^2^), and the dual PTT/chemotherapy yielded effective tumor growth suppression [81].

While the above examples demonstrate the efficacy of cytoplasmic membranes as a coating for NPs that mediate dual PTT/chemotherapy, tumor-cell-derived exosome membranes can also be used as a coating that provides homotypic tumor targeting. Tian et al*.* co-loaded porous silicon NPs with ICG and DOX and wrapped them with breast cancer-cell-derived exosome membranes [83]. The anti-tumor effect of this unique platform was tested in female BALB/c mice with orthotopic 4T1 tumors. The mice were injected with NPs on days 0 and 3, followed by tumor irradiation on days 1 and 4 (808 nm laser, 2 W/cm^2^, 4 min). Tumor temperatures reached 50 °C upon irradiation and both primary tumor growth and metastasis were inhibited through 16 days post-treatment. Collectively, the different studies presented in Sect. 4.2 make it apparent that membrane-wrapped NPs outperform their unwrapped counterparts and that combined PTT/chemotherapy is advantageous over each individual treatment, supporting the continued development of membrane-wrapped NPs for multimodal PTT/chemotherapy of cancer.

### PTT and immunotherapy mediated by membrane-wrapped NPs

#### Overview of dual PTT/immunotherapy of cancer

**Scheme 6.**
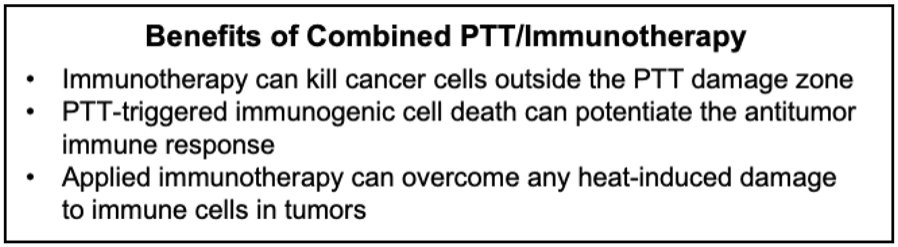
PTT and immunotherapy can each overcome the limitations of the other to provide lasting tumor regression

Cancer is difficult to treat not only because of its invasiveness and metastatic nature, but also because it can evade immune recognition. In normal physiological conditions, co-stimulatory and inhibitory signals (known as immune checkpoints) prevent autoimmunity and protect tissues when the immune system is responding to pathogenic invaders [113]. However, tumors dysregulate immune-checkpoint protein expression as an immune resistance mechanism, particularly against T cells specific for tumor-associated antigens. Fortunately, immune checkpoint receptors and ligands that are upregulated on tumor cells can be blocked by exogenously delivered antibodies or surface-modified and cargo-loaded NPs [113]. Further, these immunotherapeutic approaches can be enhanced by PTT through its ability to promote immunogenic cell death (ICD) that activates an antitumor immune response [82]. When PTT causes ICD, tumor associated antigens are released and recognized by immune cells, triggering an anti-tumor response similar to tumor vaccines. Hence, PTT can dramatically enhance immunotherapy (Scheme 6).

Conversely, immunotherapy can overcome some of the limitations of PTT as a standalone treatment. When PTT is performed alone, cancer cells outside the irradiation region can form residual lesions after treatment. Moreover, tumor hyperthermia can unintentionally damage immune cells that are required for future tumor inhibition. By combining PTT with immunotherapy, residual or metastatic tumor cells can be eliminated via immune responses and the immunotherapy can restore the immune cells in the tumor microenvironment post hyperthermia to prevent further tumor growth [15, 114]. By combining PTT-induced ICD with other immunoadjuvants or immunotherapy technologies designed into NPs, dual PTT/immunotherapy can create strong anti-tumor effects [65, 78, 82, 84, 94]. Below, examples of membrane-wrapped NPs for dual PTT/immunotherapy are introduced.

#### Membrane-wrapped NPs for PTT/immunotherapy

One potential advantage of biomimetic NPs for dual PTT/immunotherapy is that cancer cell membrane coatings surrounding NPs may be able to stimulate an anti-tumor immune response owing to their presentation of tumor antigens that result in vaccine-like effects. While membrane antigens alone may be insufficient to induce anti-tumor immune response, membrane antigens combined with other therapies like PTT, chemotherapy, and/or immune adjuvant delivery can provide robust immunotherapy [115–117]. In one example, ICG-loaded magnetic iron oxide NPs were coated with a hybrid membrane consisting of ID8 murine ovarian cancer cell membranes and RBC membranes to form Fe_3_O_4_-ICG@IRM [65]. The tumor antigens on the hybrid membrane induced an antitumor immune response after the NPs were phagocytosed by macrophages and subsequently delivered to the spleen or lymph nodes, while the RBC components enabled long circulation. Compared to NPs coated with only cancer membranes or only RBC membranes, the hybrid membrane NPs exhibited 1.7-fold and 2.1-fold increased tumor accumulation, respectively, at 8 h post IV injection based on fluorescence imaging. The Fe_3_O_4_ cores enabled magnetic field (MF)-induced therapy options as well. When ID8 subcutaneous tumor-bearing mice were IV injected with Fe_3_O_4_-ICG@IRM NPs and their tumors irradiated (808 nm laser, 1 W/cm^2^, 10 min) 8 h later, the tumor temperature increased to 54.3 °C. If MF was also applied to the irradiated tumors for 30 min, the tumor temperatures increased to 58.7 °C and the dual PTT/MF caused the greatest tumor growth inhibition with complete regression in 4 mice. Fe_3_O_4_-ICG@IRM were also tested via intratumoral injections in two types of bilateral flank tumor models (left ID8/right ID8, left ID8/right B16-F10). Twelve hours post injection, the Fe_3_O_4_-ICG@IRM NPs were mainly found in draining lymph nodes where an immune response could be activated by the tumor cell antigens in the hybrid membrane coating. Administration of Fe_3_O_4_-ICG@IRM into the left flank ID8 tumor with or without NIR irradiation did not cause any inhibitory effect on the B16-F10 tumor in the right flank. However, the ID8 tumor antigens induced specific immune responses against non-irradiated ID8 tumors in the right flank after irradiation of the left ID8 tumor. It was hypothesized that the higher accumulation of Fe_3_O_4_-ICG@IRM NPs in the spleen activated the immune response to achieve tumor-specific immunotherapy while the PTT-induced release of whole-cell tumor antigens into the tumor microenvironment also added to the antitumor immunotherapy and inhibition of primary and metastatic tumors.

When treating extremely invasive cancer types, such as basal-like breast cancer, robust and sophisticated immunotherapies are needed. Although checkpoint antibody therapies can induce a response in basal-like breast cancer, tumor recurrence rates remain high because blockade therapies do not have sufficient immunotherapeutic strength and CD8 + T cells face exhaustion by tumor cells after infiltrating the tumor. Fortunately, PTT stimulated by biomimetic membrane-coated NPs can be combined with checkpoint blockade mediated by PD-1 or CTLA-4 antibodies to reinstate CD8 + T cells’ normal activity and significantly increase immunotherapy success in basal-like breast cancer tumors [78]. This was demonstrated in a study where RBC membrane-coated black phosphorus quantum dot nanovesicles (BPQD-RMNVs) were fabricated to induce TNBC cell apoptosis via PTT. These biomimetic nanovesicles were combined with the administration of PD-1 antibodies (aPD-1) to mobilize the immune system and eliminate residual and metastatic cancer cells [78]. BPQD-RMNVs were tested in primary and “metastatic” tumors in a mouse model that was established by inoculating 4T1 breast cancer cells in the right flank (primary tumor) and then 7 days later injecting cancer cells subcutaneously in the left flank (metastatic tumor). BPQD-RMNVs were IV injected at 1 mg BP/mL into these mice 8 days after the right flank tumor inoculation and then irradiated with an 808 nm laser (1 W/cm^2^, 10 min) 2 h after injections. The aPD-1 at 5 mg/kg was IV injected on days 9, 12, 15, and 18 after initial right flank tumor inoculation. Upon NIR irradiation, BPQD-RMNVs increased tumor temperature to a maximum of 52.8 °C, which was  ~ 7 °C more than unwrapped control BPQDs. At the end of the study, statistical analysis showed that BPQD-RMNVs + laser + aPD-1 treatment (dual PTT/immunotherapy) achieved synergistic efficacy compared to aPD-1 alone (immunotherapy alone) and BPQD-RMNVs + laser without immune checkpoint blockade (PTT alone). During the therapeutic study, the left flank metastatic tumor did not receive irradiation so tumor inhibition was dependent on the CD8 + T cell activation caused by the right tumor after its irradiation. After analyzing the density of tumor-infiltrating CD8 + T cells in the left tumor from different test groups via flow cytometry and immunofluorescence, the CD8 + T cell density of BPQD-RMNVs + laser ± aPD-1 experienced a four- to five-fold increase compared to mice in the PBS control group. Moreover, the density of CD4 + T cells intensively increased in mice treated with BPQD-RMNVs + laser + aPD-1 by 37.2% and BPQD-RMNVs + laser by 18.6%. Additionally, when upregulated CD8 + T cells are exhausted, they have a reduced ability to produce immune cytokines, including IFN-ɣ and TNF-α. Impressively, BPQD-RMNVs + laser + aPD-1 treatment significantly increased serum IFN-ɣ levels in mice compared to other treatment controls. Overall, BPQD-RMNV-mediated PTT successfully caused tumor cell apoptosis which recruited dendritic cells to capture released antigens and present them to native T cells. The additional administration of aPD-1 significantly inhibited primary and secondary growth as well as stimulated a significant and beneficial immune response in vivo.

The preceding example applied checkpoint inhibitor antibodies separately from the membrane-wrapped NPs, but exogenous immunostimulatory agents can alternatively be loaded within NPs to yield dual PTT/immunotherapy. In recent work, a biomimetic nanoplatform for synergistic PTT, starvation, and immunotherapy of breast cancer was developed by loading AuNPs into dendritic mesoporous silica nanoparticles (DSMNs) and then coating the surface with a hybrid pH-sensitive membrane loaded with the immunostimulatory agent R837, fully termed AuDRM [82]. The hybrid pH-sensitive membrane was fabricated from a PEO_z_ liposome composed of lecithin, cholesterol, and DSPE-PEO_z_ then mixed with 4T1 breast cancer cell membranes and the R837. The hybrid membrane was designed to be pH-sensitive so that after arriving at the tumor, the membrane would be promptly removed to expose the AuNPs to perform starvation therapy while the R837 would be released in the tumor microenvironment to enhance immunotherapy effects. Upon irradiation, the AuNPs could also cause mild temperature elevation to induce ICD and trigger release of tumor-associated antigens, and the 4T1 membrane components could deliver tumor antigens for an even greater multi-therapeutic effect. Mild PTT was selected as a goal for this nanoplatform because extreme hyperthermia (> 55 °C) can damage antineoplastic immune cells and reduce immunotherapeutic success in the tumor microenvironment. When AuDRM at a dose of 15 mg DMSN/kg were IV injected into Balb/c mice bearing subcutaneous 4T1 tumors and allowed to circulate for 24 h, tumors reached ~ 49 °C after low intensity laser irradiation (808 nm, 0.5 W/cm^2^, 5 min). Tumors from mice treated with AuDRM + laser had the highest HSP70 expression, highest glucose-consuming efficiency, and had significantly higher immune activation based on percent infiltration of mature dendritic cells and proliferous T cells compared to other treatment groups. AuDRM + laser also caused 100% tumor inhibition by ~ 15 days post treatment and kept mice alive for 80 days to the end of the study, whereas other treatment groups experienced tumor growth and death by day 45. When antimetastatic efficacy was evaluated in a model established by IV injecting luciferase-expressing 4T1 cells, mice treated with AuDRM + laser had the least metastases and highest overall survival compared to other treatment groups. After treatments, the tumors were resected and the mice reinoculated with 4T1 cells 28 days later to assess the immune response and long-term memory established by the treatments. Mice with previous AuDRM + laser treatment experienced elevated percent of effector memory T cells, increased CD8 + cells in distant tumors, significant inhibition of distant 4T1 tumors, and survival until day 50, whereas mice from other treatment groups gradually died within 40 days. This synergistic nanoplatform successfully inhibited primary tumor growth, served as a holistic treatment of metastases, and induced long-term immune memory effect to reduce tumor recurrence and prolong survival. The PTT-induced immunogenic tumor microenvironment combined with the immunostimulatory R837 created a vaccine-like function against primary tumors as well as immunotherapy against metastases and future tumor relapse.

#### Membrane modifications for enhanced PTT/immunotherapy

Not only can immunostimulatory or other exogenous immunotherapeutic agents be loaded within or injected along with biomimetic NPs to enhance combination PTT/immunotherapy, but cell membrane coatings on NPs can be primed or pre-engineered to facilitate activation of dendritic cells and T cells for enhanced antitumor immune responses. Xu et al. utilized a semiconducting polymer nanoengager (SPNE) coated with a hybrid membrane composed of 4T1 breast cancer cell membranes and dendritic cells (DCs) that were pre-engineered to have enhanced levels of damage-associated molecules patterns (DAMPs) and T cell stimulating-factors, respectively [84]. The SPNE core, synthesized with poly(benzobisthiadiazole-alt-thiophene), is a highly NIR-II absorbing material that can mediate PTT and induce ICD. To pre-engineer the hybrid membrane components prior to extraction, 4T1 cells were pre-incubated with doxorubicin and DCs were stimulated with resiquimod (R848) and tumor antigens. To test combination PTT/immunotherapy in vivo, bilateral tumors were established by subcutaneous inoculation of 4T1 cells onto the right and left flanks of Balb/c mice with 6 days in between inoculations. After IV injections of SPNE and uncoated SPN (SPNU) followed by 1064 nm laser irradiation (1 W/cm^2^, 10 min) of primary tumors 24 h post injection, SPNE + laser caused 97% primary tumor inhibition with negligible primary tumor recurrence compared to accelerated tumor growth in SPNU-treated mice by 11 days after irradiation. Additionally, SPNE + laser treated mice exhibited 85% inhibition in growth of distant tumors on opposite flanks as well as reduction in pulmonary and liver metastatic nodules which confirms the potency of SPNE-mediated PTT immune responses. SPNE + laser treated tumors showed the highest expression of both the apoptosis marker caspase-3 and the hallmark of ICD, high mobility group box 1 (HMGB1), compared to other test groups. The results show SPNE-mediated PTT/immunotherapy synergized the vaccination effect from the membrane shell with the ICD effect from PTT, stimulating superior systemic anti-tumor responses over singular PTT or immunotherapy. Additionally, the long-term antitumor immunity was compared between cancer mouse models and healthy mice and tests confirmed that immunological memory could be established by SPNE-mediated PTT immunotherapy.

As an alternative to pre-engineering membranes to express distinctive antigens, bacterial outer membrane vesicles (OMVs) can be incorporated into NPs’ membrane exterior to enhance immunotherapeutic success. When bacterial OMVs accumulate in tumor tissue they induce the production of antitumor cytokines and stimulate DC maturation [94]. Researchers have exploited this by fusing the OMV derived from E. coli DH5α with B16-F10 cancer cell membranes to produce OMV-cancer cell hybrid membranes that were coated onto hollow polydopamine (HPDA) NPs to obtain HPDA@[OMV-CC] NPs [94]. When tested in vivo, the OMV addition to the hybrid coating was proven to increase IL-12p40 and interferon (IFN)-γ cytokine generation in blood serum and tumors compared to cancer cell membrane-only wrapped HPDAs. When tumors of HPDA@[OMV-CC] NP-treated mice were irradiated with a 1064 nm laser four-hour post-injection (1 W/cm^2^, 10 min) they reached a maximum temperature of 52.3 °C and experienced tumor growth inhibition of  ~ 99%, whereas cancer cell membrane-only wrapped HPDAs inhibited tumor growth by only  ~ 68.5%. Without laser irradiation, HPDA@[OMV-CC] NPs still had a tumor growth inhibition of about 31.7% demonstrating the immunotherapy potential of the NPs alone. To assess long-term immune memory, tumors from all treatment groups were removed after 13 days and mice rechallenged with cancer cells. Over 80% of mice previously treated with HPDA@[OMV-CC] NPs with laser irradiation experienced tumor disappearance with survival up to 45 days (the end of the study).

The papers discussed in this section show that both pre-engineering cells to present specific antigens or incorporating bacterial OMVs into hybrid membrane coatings are valuable strategies towards creating synergistic PTT immunotherapies with the capabilities to destroy primary tumors and prevent recurrence upon rechallenge. More broadly, the studies summarized in Sect. 4.3 show that membrane-wrapped NPs enabling dual PTT and immunotherapy can synergistically shrink primary tumors and distant metastases while priming the immune system to prevent recurrence after the initial disease is eliminated. PTT and immunotherapy utilizing biomimetic NPs are mutually beneficial to one another and help enhance each treatment’s short- and long-term anticancer effects. PTT aids immunotherapy by causing ICD and recruitment of immune cells to the irradiated location where they can engulf NPs and any immunostimulatory cargo they contain. Immunotherapy can overcome the obstacles faced by PTT alone, such as residual tumor lesions and inability to treat disseminated disease. The mutually beneficial relationship between PTT and immunotherapy warrants further development of combination treatments mediated by membrane-wrapped NPs, as this approach offers remarkably strong and long-lasting anti-tumor effects.

### Dual PTT and radiotherapy mediated by membrane-wrapped NPs

**Scheme 7.**
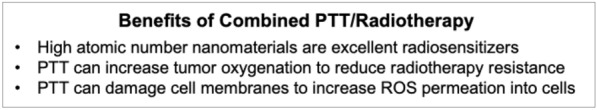
Overview of the benefits of combined PTT and radiotherapy

In relatively new developments, membrane-wrapped NPs enabling PTT are being combined with radiotherapy (RT), one of the longest-used clinical treatments for solid tumors [67]. In RT, tumors are exposed to high-energy X-ray or ionizing radiation (IR), which produces free radicals that induce DNA damage leading to cell death [41]. Despite RT having no tissue depth restriction, the dose of IR is strictly regulated in the clinic to minimize side effects in non-target tissues which unfortunately attenuates the therapeutic effect in targeted tumors. Additionally, RT suffers from cellular resistance due to hypoxia in the tumor microenvironment [41]. To overcome dose limitations and resistance mechanisms, phototherapeutic NPs are being used to enhance RT by serving as exogenous energy absorbers that increase free radical production and/or by inflicting damage to the tumor microenvironment that increases intratumoral blood flow and oxygenation (Scheme 7) [41, 67]. Overall, combining RT with PTT can yield an enhanced and sometimes synergistic effect against solid tumors.

High-Z nanomaterials are most appropriate for RT enhancement because photoelectric interactions are positively correlated with atomic number [67]. Bismuth, gold, and copper sulfide (CuS) NPs have all been wrapped with cell membranes and explored as dual PTT mediators and radiosensitizers owing to their high atomic number [41, 67, 90]. In one study, bismuth sulfide nanorods (BNRs) were prepared and decorated with mesoporous silica to allow linkage of fluorescent probes for material tracing [67]. Platelet membranes were then coated onto the BNRs to camouflage them and endow tumor targeting capabilities (BMSNR@PM). The BMSNR@PM were administered IV at 25 mg/kg into Balb/c mice bearing subcutaneous 4T1 tumors, and 6 h later the tumors were irradiated with 808 nm light (1 W/cm^2^, 10 min) and given a local low dose of 5 Gy X-ray irradiation (1.0 Gy/min). By three weeks post treatment, the dual PTT/RT mediated by BMSNR@PM significantly lowered the tumor mass and volume compared to NP-mediated PTT or RT alone. An isobologram analysis demonstrated a synergistic effect of BMSNR@PM-based PTT on RT in vivo. It was assumed that the extracellular matrix destruction and promotion of intratumoral oxygenation caused by PTT was the main cause for the enhanced RT effects*.*

RBC-membrane wrapped CuS NPs and oral squamous cancer cell membrane-wrapped gold nanorods have also been explored for dual PTT and RT enhancement [41, 90]. The RBC-CuS NP system was only evaluated in vitro, but dual PTT and RT mediated by this system decreased 4T1 breast cancer cell viability by 86% compared to 54% for NP-mediated PTT alone or 30% for NP-mediated RT alone [90]. Compared to copper sulfide and other high-Z nanomaterials, gold-based NPs offer the advantage of increased biocompatibility while maintaining desirable optical properties and radiosensitization abilities [41]. Since RT is commonly used to treat oral squamous cancer in the clinic, gold nanorods with longitudinal SPR in the NIR-II window were synthesized and coated with oral squamous KB cell membranes (GNR@Mem) for combined PTT and RT of oral squamous cancer [41]. The GNR@Mem were tested in nude mice with flank KB tumors. GNR@Mem or a PEG coated control (GNR@PEG) were IV injected at 5 mg/kg and then tumors irradiated with a 980 nm laser (0.5 W/cm^2^, 5 min) and/or 4 Gy X-ray. GNR@Mem exhibited blood clearance half-life of 4.36 h compared to only 2.71 h for GNR@PEG. When combined with NIR laser and X-ray irradiation, GNR@Mem destroyed 4 of 5 tumors in the test group and caused a 95.6% tumor volume suppression rate, which was the best tumor inhibition compared to all GNR@PEG or single irradiation controls. This study, along with those discussed above, corroborates that dual PTT and RT mediated by membrane-wrapped NPs holds great promise as a cancer treatment strategy.

### Combined PTT and photodynamic therapy enhanced by membrane-wrapped NPs

**Scheme 8.**
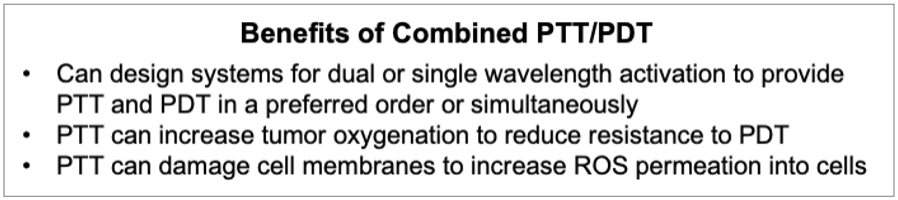
Summary of benefits of dual PTT/PDT

Photodynamic therapy (PDT) is a light-activated cancer therapy that nicely complements PTT since it inhibits tumor growth by using light to activate photosensitizers leading to generation of singlet oxygen (^1^O_2_) and other reactive oxygen species (ROS) that induce cellular damage [58]. Several biomimetic NP designs have combined PTT with PDT exclusively or in combination with other therapeutic approaches or imaging strategies, including those based on gold [45], zinc phthalocyanine (ZNPC) [80], Prussian blue [56, 58], semiconducting polymers [96], graphene oxide quantum dots [76], tungsten disulfide nanosheets [63], and liposomes [73]. Usually PDT and PTT agents are activated by light of different wavelengths, so researchers must carefully determine the appropriate timing and dosage of each agent and their respective activating light sources to cause the greatest tumor ablation. Recently, the hydrophobic photosensitizer ZNPC was co-assembled with ICG and loaded into RBC membranes to create a biomimetic NP capable of single NIR laser-induced PTT/PDT at 680 nm [80]. The ZNPC-ICG@RBC nanoprobes simultaneously produced ROS and heat to synergistically eliminate tumors and prevent cancer regrowth as determined by fluorescent imaging analyses. These results demonstrate the feasibility of producing nanoprobes that can support single wavelength activated dual PTT/PDT, and the potential impact of such a treatment approach (Scheme 8).

Another issue with dual PTT/PDT is the limited biocompatibility of photoactive agents along with their lack of functional groups and poor bioavailability. For example, Chlorin e6 (Ce6) is a promising photosensitizer with high ^1^O_2_ quantum yield but it suffers from rapid blood clearance and aggregation [58]. To address these shortcomings, Ce6 was embedded in RBC membrane vesicles via its lipophilic fractions’ strong affinity for the RBCs’ lipid membranes, and then the Ce6 modified RBC membranes were coated onto Prussian Blue NPs (PB@RBC/Ce6 NPs) [58]. When irradiated with a 660 nm laser, the PB@RBC/Ce6 NPs membrane components disintegrated quickly, and a Ce6-mediated PDT effect was achieved. Subsequent application of 808 nm light activated the PB NP cores to achieve PTT. This design stabilized Ce6 to increase its blood circulation and accumulation in orthotopic 4T1 tumors in mice. This NP design shows promise for future dual PTT/PDT biomimetic NP designs as it inspires the integration of natural biological components (i.e., cell membranes) with synthetic nanomaterials (i.e., Ce6 and other PDT or PTT photosensitizers) to create photoactive therapeutics with improved bioavailability, cellular endocytosis, and antitumor effects.

Notably, incorporating gold NPs into dual PTT/PDT designs can offer some of the same benefits as the systems discussed above while also enabling tumor imaging. In one example, Sun et al. loaded ICG in gold nanodendrites (AuND) to provide PTT, PDT, photoacoustic imaging, fluorescence imaging, and surface enhance Raman scattering (SERS) imaging [45]. The AuNDs were coated with RAW 264.7 macrophage membranes to enable targeted delivery to MDA-MB-231 human breast cancer cells via the active binding of macrophage-expressed α4 integrins to overexpressed vascular cell adhesion molecule-1 (VCAM-1) on MDA-MB-231 s. The AuNDs were also conjugated with a mitochondria-targeting cationic compound, triphenylphosphonium (TPP), to help deliver the ICG specifically to mitochondria once inside target cells to increase PDT efficacy, and this also enabled SERS imaging. The complete formulation was termed AuND-TPP-ICGs@MCM. When IV injected into MDA-MB-231 tumor-bearing mice, the AuND-TPP-ICG@MCM successfully enabled fluorescent imaging (Ex: 640 nm, Em: 720 nm), NIR-II photoacoustic imaging (Ex: 1200 nm via a visual sonic system), and SERS-based Raman imaging (785 nm diode laser). The NPs also successfully mediated PTT and PDT when tumor-bearing mice received a total of 3 IV injections of AuND-TPP-ICG@MCM or controls every 2 days followed by 1064 nm (1.0 W/cm^2^, 10 min) and/or 808 nm (0.3 W/cm^2^, 5 min) laser irradiations 12 h after each injection. The greatest tumor ablation and most pronounced level of ^1^O_2_ production was observed in mice treated with AuND-TPP-ICG@MCM combined with both 1064 and 808 nm laser irradiation. This unique system impressively demonstrates the ability to provide NIR-II PTT and NIR-I PDT along with fluorescence, photoacoustic, and SERS imaging in a single platform.

Finally, semiconducting polymer nanoparticles (SPNs) are another promising platform for combined PTT/PDT and imaging. SPNs possess excellent photonic properties that allow them to support NIR fluorescence, chemiluminescence, photoacoustic imaging, and phototherapy, among other capabilities [96]. In one elegant study, Li et al. wrapped activated fibroblast membranes around poly-(cyclopentadithiophene-alt-benzothiadiazole) to create a biomimetic SPN (AF-SPN) capable of multimodal cancer phototheranostics [96]. The activated fibroblast membrane wrapping was a unique and sophisticated approach to perform homologous targeting of cancer-associated fibroblasts in the tumor stromal microenvironment, which are key contributors towards creating the physical barrier that impedes NP delivery deep within tumors. To test the effectiveness of AF-SPNs, unwrapped SPNs (uSPNs) and 4T1 murine breast cancer cell membrane wrapped SPNs (CC-SPNs) were used as controls. Importantly, the absorbance characteristics, fluorescence intensity, and photoacoustic signals of the SPNs remained unchanged upon membrane wrapping and the photothermal conversion efficiency of CC-SPNs and AF-SPNs excited with an 808 nm laser were 27.7 and 26.7%, respectively, similar to that of uSPNs (26.3%) [96]. Following in vitro studies that validated the improved uptake of membrane-wrapped SPNs versus uSPNs in activated fibroblast and 4T1 cell cultures, in vivo studies were performed wherein AF-SPNs and controls were IV administered to nude mice bearing 4T1 tumors. Forty-eight hours post injection, the tumor fluorescence intensity of AF-SPN treated mice was 1.5- and 1.3-fold higher than that of uSPN and CC-SPN injected mice, respectively. Correspondingly, the maximal PA intensity enhancement (ΔPA) in AF-SPN treated mice was 1.8- and 1.5-fold higher than that for uSPN and CC-SPN treated mice, respectively. To test phototherapeutic efficacy, mice were exposed to 808 nm laser irradiation (0.3 W/cm^2^ for 5 min) forty-eight hours post IV treatment administration. The maximal tumor temperature was 50 °C for AF-SPN injected mice, which was 4.0, 6.0, and 14.0 °C higher than that for CC-SPN, uSPN, and saline injected mice, respectively. Congruently, tumor volumes decreased to a greater extent in mice that received irradiation plus AF-SPN treatment compared to uSPN, CC-SPN, and saline controls. Post-mortem analysis of excised tumors confirmed that the tumor response was mediated by PDT in addition to PTT. Overall, this study confirmed that targeting activated fibroblasts with membrane-wrapped SPNs is a promising approach to enhance cancer imaging and phototherapy. In aggregate, the studies discussed in this section demonstrate that dual PTT/PDT mediated by membrane-wrapped NPs is a therapeutic approach with great potential against solid tumor cancers.

## Conclusions: remaining challenges and the path forward for membrane-wrapped NPs for PTT

This review highlights the current state-of-the-art in cell-membrane camouflaged NPs for solid-tumor cancer PTT. Effective PTT requires photothermally-active NPs to accumulate at the tumor site in quantities that are sufficient to increase temperatures above 42 °C upon NIR irradiation to kill cancer cells [32]. One challenge is that photoresponsive NPs (like other nanomaterials and small drugs) are quickly recognized by the immune system as foreign invaders and are cleared from circulation, limiting tumor accumulation. Camouflaging photosensitive NPs with cell membranes does not alter their optical properties or heating abilities [44, 46, 91], and endows them with unique properties of the source cells. These properties include immune evasion abilities that prolong systemic circulation and improve tumor delivery through passive accumulation or homotypic targeting [118]. Hence, membrane wrapping can help ensure photoresponsive NPs arrive at tumor sites in adequate therapeutic doses [118]. To further increase tumor targeting, source cell membranes can be engineered to express tumor-targeting moieties or functionalized with antibodies or other ligands specific to receptors that are overexpressed on the target cancer cells [27, 119]. Such approaches remain to be refined and improved but have great promise.

Importantly, membrane-wrapped NPs for PTT must not only be effective in eliciting tumor heating above the damage threshold, but also have a reasonable safety profile such that their efficacy substantially outweighs any potential toxicity. It is hence imperative that researchers confirm both the NP core and the membrane coating are independently biocompatible. This biocompatibility can be assessed through histological methods, evaluation of serum cytokines and liver enzymes, hemolysis assays, complete blood count tests, as well as other techniques that are common in preclinical and clinical studies [111, 120, 121]. Current research indicates minimal toxicity of membrane-wrapped NPs, but thorough analysis will be required before these systems can move forward to human clinical testing and use.

Excitingly, membrane-wrapped NPs can enable not only PTT, but also stimuli-responsive release of encapsulated drugs or immunostimulatory agents to maximize tumor inhibition in combined treatment strategies [30]. PTT has been limited by the shallow penetration depth of laser light in tissues in vivo, making it ineffective in eradicating deep-tumors. Combining PTT with other therapies, or developing photosensitizers that absorb light in the NIR-II window rather than the NIR-I window, can help overcome this limitation [32, 100]. This has been demonstrated as feasible in literature, where PTT has been combined with chemotherapy, PDT, immunotherapy, and RT with great success [41, 45, 84, 94]. Compared to PTT alone, which may effectively treat only primary tumors, studies have shown that membrane-wrapped NPs enabling PTT along with other therapies can inhibit primary tumors, metastases, and even prevent recurrence [68, 84, 94]. In particular, PTT + chemotherapy and PTT + immunotherapy are amenable to the treatment of metastatic disease [40, 65, 78, 82–84, 95], while PTT + immunotherapy is capable of preventing recurrence [82, 84]. This is a major advantage over the combination of PTT with RT or PDT, which may effectively eliminate solid tumors, but not disseminated disease. Further exploration into multimodal therapies mediated by membrane-wrapped NPs will be an important direction for the field.

It is worth noting that the anti-tumor efficacy of PTT is determined by many factors including laser power density, irradiation time, and frequency, and that the appropriate level of these will be influenced by the concentration of NPs within the tumor. Membrane-wrapped NPs that encapsulate contrast agents or have inherent contrast enhancing features can support real-time imaging to ensure that enough NPs arrive within tumors to mediate PTT [45, 96, 98]. Additionally, thermal imaging techniques such as magnetic resonance thermal imaging could be further developed and used to ensure that the tumor has reached ample temperature for a sufficient length of time, as demonstrated in previous animal and human studies [5, 122]. Researchers have demonstrated that membrane-wrapped NPs can enable PTT along with MRI, photoacoustic imaging, fluorescence imaging, and other imaging modalities that vary based on the specific NP design [61, 62, 64, 72]. The continued development and application of image-guided PTT will help this technology achieve clinical success.

Before cell membrane-wrapped NPs for PTT can be translated to the clinic, various items need to be addressed. While these systems have shown good biocompatibility in short-term animal studies, their long-term safety needs to be carefully investigated since membrane-wrapped NPs accumulate in healthy organs like liver and spleen besides tumor tissues [30]. Additionally, quality-control and stringent testing procedures must be developed to ensure the extracted membrane vesicles are pure and free of contaminants like pathogens or viruses or any genetic material to avoid any oncological possibility [32, 34]. Any portions of denatured proteins in the cell membrane vesicles must be eliminated to avoid immune responses against endogenous antigens [109, 119]. As the development of cell membrane-wrapped photoresponsive NPs is maturing, these issues should be overcome and the shift from discovery to process development could be realized quickly.

**Scheme 9.**
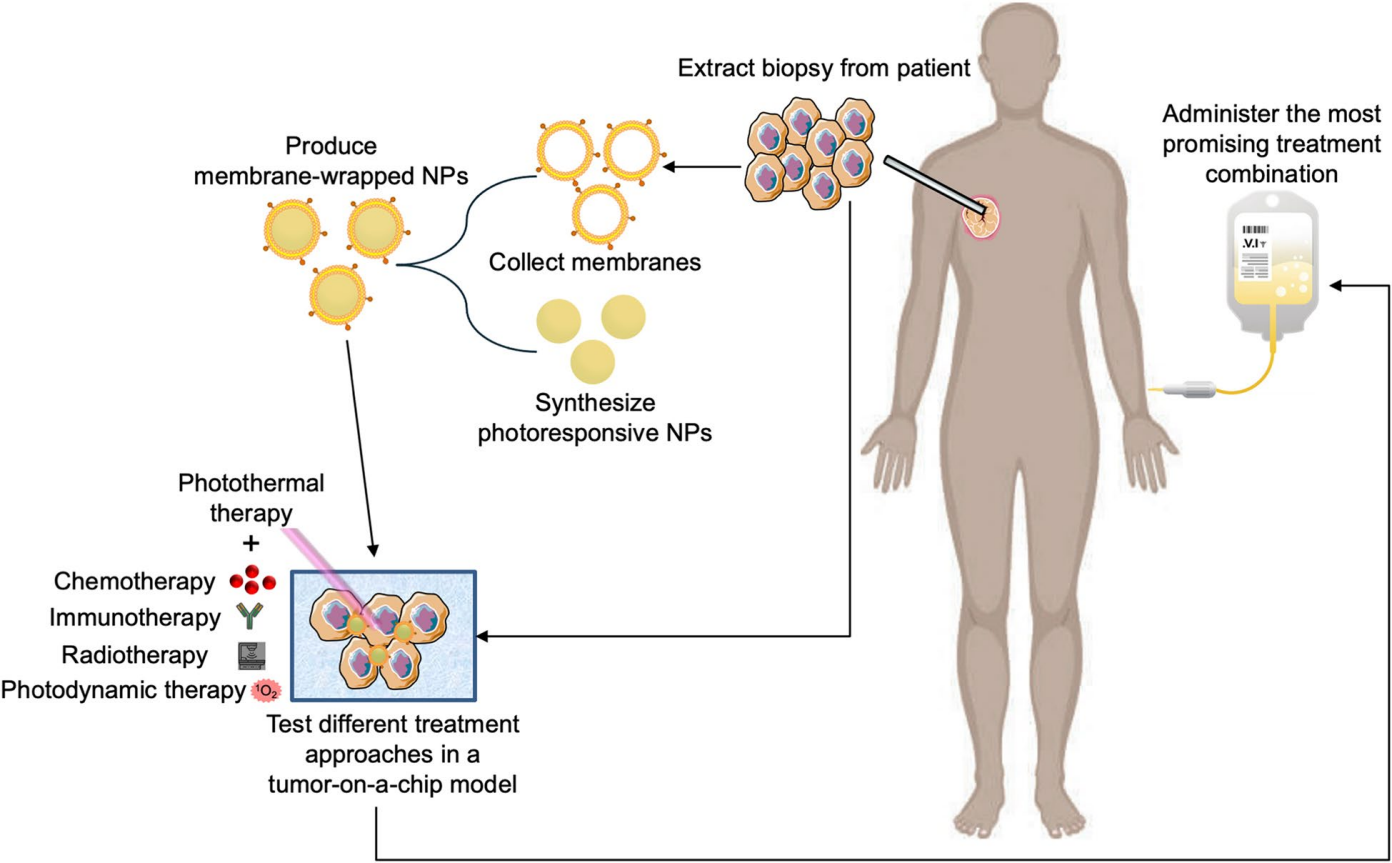
Scheme depicting a vision for the future development of personalized therapies based on phototherapeutic membrane-wrapped NPs. Individual patient’s own cancer cells (obtained from a biopsy) could be used to create both membrane-wrapped NPs and tumor-on-a-chip models that would be implemented to test the efficacy of PTT alone or in combination regimens. The most effective strategy identified in the model could then be administered to the patient. Portions of this figure were produced using Servier Medical Art templates (https://smart.servier.com). Servier Medical Art by Servier is licensed under a Creative Commons Attribution 4.0 Unported License

One major advantage of membrane-wrapped photoresponsive NPs is the vast opportunity to make personalized treatments by using a patient’s own cells to avoid an immune response. If making NPs for each individual patient is not feasible, using donor cell membranes could be a solution, but one should be aware of potential immunostimulatory issues. To minimize the amount of foreign material that might induce an immune response, hybrid membranes made from a mixture of donor cancer cells and the patient’s own RBCs or platelets could be used [34]. Other membrane sources like stem cells could be challenging to implement because they are rare in the body; likewise, use of donor RBCs or leukocytes would require blood-type compatibility [118]. In general, the membrane source should be carefully selected when developing biomimetic NPs to ensure patient compliance, maximize therapeutic effect, and minimize immune response. For personalized therapies to be maximally successful, one might envision a future where a patient’s own tumor cells (collected from a biopsy) would be used both for NP production and generation of personalized tumor-on-chip models (Scheme 9). This would allow membrane-wrapped NPs carrying distinct cargo to be tested in the chip to identify the best therapeutic approach (i.e., PTT applied alone or in combination with chemotherapy, immunotherapy, RT, or PDT) for a specific patient prior to administering the most effective mono or combination therapy in the clinic (Scheme 9). Such strategies are bold and challenging, yet also achievable given recent advancements in the field.

Lastly, for cell membrane-wrapped phototherapeutic NPs to be successful clinically, manufacturing scale-up needs to be developed and optimized. The process of membrane extraction needs to be improved to minimize material loss [118]. Different membrane extraction and wrapping methods have been reported [34], but the parameters are varied and there is currently no basis for judgment on which method is better. Moreover, batch-to-batch consistency for multi-component biomimetic phototherapeutic NPs needs to be addressed before scaling up. Clinical doses of membrane-wrapped NPs will require huge amounts of membranes to be extracted (which is time-consuming and labor-intensive), and large volumes of NP cores will need to be produced. The process of fusing membrane vesicles with NPs will need to be optimized for large-scale production. Microfluidic electroporation devices may offer one strategy to commercialize the fusion process [62]. Finally, good manufacturing practices (GMPs) and good laboratory practices (GLPs) must be established to ensure quality standards are met and improve the rate of translating nanomedicine from bench to bedside.

Once the issues discussed here are addressed, PTT mediated by membrane-wrapped NPs will have great potential to transform cancer patient care. Each patient’s particular condition will have to be taken into careful consideration when designing biomimetic NPs for PTT and selecting which other treatment modalities to apply in combination with this therapy. The future of biomimetic NPs for PTT lies in solving these issues to achieve precise thermal ablation of solid-tumor cancers while providing specific, personalized, and maximally effective therapy for individual patients.

## Data Availability

The data discussed in this review are available from the original articles referenced.

## Abbreviations

PTT: Photothermal therapy
NPs: Nanoparticles
IV: Intravenously
EPR: Enhanced permeability and retention
MPS: Mononuclear phagocytic system
PEG: Polyethylene glycol
ABC: Accelerated blood clearance
PLGA: Poly(lactic-co-glycolic acid)
RBC: Red blood cell
NIR: Near-infrared
ICG: Indocyanine green
MRI: Magnetic resonance imaging
MSCs: Mesenchymal stem cells
SDF-1: Stromal cell-derived factor-1
MHC: Major histocompatibility complex
MDSCs: Myeloid derived suppressor cells
CAR-T cells: Chimeric antigen receptor T cells
SPR: Surface plasmon resonance
TNBC: Triple-negative breast cancer
AuNCs: Gold nanocages
PVP: Poly(vinylpyrrolidone)
AuNRs: Gold nanorods
PLT-M-AuNRs: Platelet membrane-wrapped AuNRs
GPC3: Glypican-3
EpCAM: Epithelial cell adhesion molecule
PDA: Polydopamine
PCL: Poly(caprolactone)
SN38: 7-Ethyl-10-hydroxycamptothecin
H40-PEG: Hyperbranched PEG
TPZ: Tirapazamine
Asp8: Aspartic acid octopeptides
PTX: Paclitaxel
DPPC: 1,2-Dipalmitoyl-sn-glycero-3-phosphocholine
DOX: Doxorubicin
2D: Two-dimensional
FA: Folic acid
DACHPt: (1,2- Diaminocyclohexane) platinum (II)
WS_2_: Tungsten disulfide
MOFs: Metal organic frameworks
HA: Hyaluronic acid
ZGGO: Zn_1.25_Ga_1.5_Ge_0.25_O_4_:Cr^3+^,Yb^3+^,Er^3+^
10-HCPT: 10-Hydroxycamptothecin
MF: Magnetic field
aPD-1: PD-1 antibodies
BPQD-RMNVs: RBC membrane-coated black phosphorus quantum dot nanovesicles
DSMNs: Dendritic mesoporous silica nanoparticles
SPNE: Semiconducting polymer nanoengager
DCs: Dendritic cells
DAMPs: Damage-associated molecules patterns
HMGB1: High mobility group box 1
OMVs: Outer membrane vesicles
HPDA: Hollow polydopamine
IFN: Interferon
RT: Radiotherapy
IR: Ionizing radiation
CuS: Copper sulfide
BNRs: Bismuth sulfide nanorods
PDT: Photodynamic therapy
^1^O_2_: Singlet oxygen
ROS: Reactive oxygen species
ZNPC: Zinc phthalocyanine
Ce6: Chlorin e6
AuND: Gold nanodendrites
SERS: Surface enhance Raman scattering
VCAM-1: Vascular cell adhesion molecule-1
TPP: Triphenylphosphonium
SPNs: Semiconducting polymer nanoparticles
PAI: Photoacoustic imaging
FI: Fluorescence imaging
MRI: Magnetic resonance imaging
CT: Computed tomography
ADME: Absorption, distribution, metabolism, elimination
GMP: Good manufacturing practice
GLP: Good laboratory practice
